# Androgen-induced AR-BRD4 transcriptional regulatory complex promotes malignant proliferation of osteosarcoma cells

**DOI:** 10.1038/s41420-025-02541-6

**Published:** 2025-06-10

**Authors:** Jia-Ming Tian, Yi-He Dong, Zi Li, Yong Zhou, Jun Huang

**Affiliations:** 1 Department of Orthopedics, The Second Xiangya Hospital, Central South University, Changsha, Hunan China; 2 Department of Orthopedics, The Affiliation Hospital of Yiyang Medical College, YiYang, Hunan China

**Keywords:** Bone cancer, Gene regulation

## Abstract

Osteosarcoma (OS) is deemed as hormone-dependent neoplasm. Here we explored its latent mechanisms governing the interactions between specific molecules and hormones involved in OS progression. Through multiplex IHC analysis in TMA, bioinformatics analysis, and a series of in vitro and in vivo molecular assays, we identified BRD4 and sex steroid receptors were positively expressed in clinical OS tissues, simultaneously BRD4 and AR expression were elevated in the osteoblastic subtype, while ERβ predominated in the fibroblastic subtype. GEO database revealed a positive correlation between BRD4 and AR expression, while no correlation was found with ERβ expression. In vitro studies demonstrated that DHT stimulation resulted in a significant upregulation of AR and BRD4 protein expression, subsequently promoting the proliferation of OS cells. ChIP-sequencing and dual-luciferase reporter assays revealed that DHT treatment increased the distribution of BRD4 on chromatin and its overlap with AR, facilitating the formation of the AR-BRD4 transcriptional regulatory complex, which significantly increased transcription levels of AR target genes, such as PLCB4. Moreover, experiments conducted in nude mice indicated that BRD4 inhibitor, (+)-JQ1 decreased the expression of AR-related genes and inhibited OS cell growth in vivo. In conclusion, elevated expression of BRD4 in OS cells induced by androgens participates in AR-related transcriptional regulatory processes, facilitating the malignant progression of OS.

## Introduction

Osteosarcoma (OS) is the most common malignant primary bone tumor and is associated with a poor prognosis in children and young adults [1]. Over the past four decades, the standard treatment regimen for OS, which includes surgical intervention and chemotherapy, has failed to adequately address critical challenges such as recurrence, metastasis, and drug resistance, leading to stagnant overall survival rates [2, 3]. Therefore, advancing adjuvant therapeutic strategies for OS requires a multifaceted approach focused on identifying novel insights and potential targets to develop effective intervention for OS patients.

OS is a hormone-dependent malignancy characterized by distinct gender- and age-related features in its onset and prognosis [4]. It predominantly affects adolescents and young individuals post-puberty, with a higher incidence in males and a comparatively poorer prognosis than in females [5]. The hypothesis linking skeletal growth and development during puberty to the onset of OS was first proposed in the 1960s [6], sparking global interest and research into the hormonal etiology of this malignancy. However, the rarity and heterogeneity of OS pose significant challenges in obtaining robust analytical evidence to substantiate its hormonal origins.

Bromodomain-containing protein 4 (BRD4), a member of the bromodomain and extraterminal (BET) protein family, plays a critical role in regulating gene transcription by recognizing highly acetylated chromatin. This function influences a variety of biological processes closely linked to tumor initiation and progression [7]. As a key anti-cancer target, BRD4 has been implicated in the pathogenesis of multiple hormone-dependent malignancies, such as ovarian [8, 9], breast [10–12], and prostate [13] cancers, especially in highly heterogeneous and invasive cancer subtypes. However, the interaction between sex steroids and BRD4 in OS remains unexplored in the current literature. Retrospective studies have shown a significant association between elevated BRD4 expression and reduced short-term survival in OS patients [14], indicating that BRD4 may represent a promising therapeutic target for OS and merits further investigation.

In this study, we aimed to elucidate the hormonal factors contributing to the poor prognosis of OS and to investigate the role of BRD4 in this context. Initially, we analyzed the expression levels of BRD4, androgen receptor (AR), estrogen receptor (ER) α, ERβ, and aromatase in patient-derived cancer tissue using a tissue microarray (TMA). Subsequently, we evaluated the effects of the active forms both of androgens (dihydrotestosterone, DHT) and estrogens (17β-estradiol, E2) forms on the malignant proliferation of OS cells. Mechanistically, we investigated how androgens influence BRD4 expression and their dynamic epigenetic regulatory mechanisms. In summary, our findings enhance our understanding of the molecular mechanisms through which sex steroids influence the development of OS and suggest that BRD4 is a promising target for disrupting androgen signaling’s supportive role in OS malignant proliferation.

## Results

### BRD4, AR, and ERβ exhibit relatively high positive rates in the tissues of OS patients

Sex-based discrepancies in cancer incidence, including OS, have been observed in nearly all human cancers, however, the underlying mechanisms responsible for these sex differences remain poorly understood [15]. Therefore, we used mIF technology to analyze the expression levels of BRD4 and sex hormone receptors, including ERα, ERβ, and AR, in OS samples. Additionally, we measured the expression levels of aromatase, the enzyme responsible for converting androgens into estrogens in vivo. After excluding specimens with missing data, 57 samples were included in the analysis. Notably, the positive rates for BRD4, AR, and ERβ were relatively high at 73.68%, 84.21%, and 56.00%, respectively. In contrast, the positive rates for ERα and aromatase were significantly lower, recorded at 36.84% and 15.79%, respectively (Supplementary Fig. 1A–D and Table 1). It is important to note that any observable fluorescence in the tissues, regardless of its intensity, was classified as a positive detection. The findings suggest that OS exhibits characteristics of a hormone-sensitive tumor, indicating that AR and ERβ may be significant targets for further investigation.

**Table 1:** Numbers of staining results for various indicators.

Staining	None	Low	Middle	High	Total	Positive rate (%)
BRD4	15	13	11	18	57	73.68
AR	9	34	14	0		84.21
ERa	36	21	0	0		36.84
ERβ	25	28	4	0		56.00
Aromatase	48	9	0	0		15.79

However, we found no statistically significant associations between BRD4 or AR expression and gender, age, tumor, grade, or TNM stage (Tables 2–4). We speculate that this may be due to limitations in OS tissue sample collection. Specifically, limited sample size restricted our ability to gather statistical evidence on the correlation between BRD4 or AR expression and OS with lymph node or distal metastasis, as well as relationships among OS subtypes. Additionally, biopsy is not a mandatory in OS diagnosis and surgery is often performed after adjuvant chemotherapy, some of the 57 samples analyzed were from patients who had already undergone treatment, and the impact of these treated samples on our analysis remains unclear.

**Table 2:** Correlation analysis of BRD4 expression level with patient’s age,gender,tumor stage and grade.

	BRD4 expression		*P*-value (two-tailed)
	High/middle	Low/Negative	
*Ages,year*			
≤25	22/38 (57.89%)	16/38 (42.15%)	0.17
>25	7/19 (36.84%)	12/19 (63.15%)	
*Gender*			
Male	20/37 (54.05%)	17/37 (45.94%)	0.59
Female	9/20 (45.00%)	11/20 (55.00%)	
*Tumor grade*			
Stage I/II	22/44 (50.00%)	22/44 (50.00%)	1.00
Stage III/IV	7/13 (53.85%)	6/13 (46.15%)	
Tumor size (T)			
T1–T2	22/39 (56.41%)	17/39 (45.59%)	0.26
T3	7/18 (38.89%)	11/18 (61.11%)	
Lymph nodes metastasis (N)			
N0	28/56 (50.00%)	28/56 (50.00%)	Count < 5
N1	1/1 (100%)	0/1 (0%)	
Metastasis (M)			
M0	25/50 (50.00%)	25/50 (50.00%)	1.00
M1	4/7 (57.14%)	3/7 (42.86%)

**Table 3:** Correlation analysis of AR expression level with patient’s age,gender,tumor stage and grade.

	AR expression		*P*-value (two-tailed)
	High/Middle	Low/Negative	
*Ages,year*			
≤25	12/39 (30.77%)	27/39 (69.23%)	1.00
>25	5/18 (27.78%)	13/18 (72.22%)	
*Gender*			
Male	9/37 (24.32%)	28/37 (75.68%)	0.54
Female	7/20 (35.00%)	13/20 (65.00%)	
*Tumor grade*			
Stage I/II	12/44 (27.27%)	32/44 (72.73%)	1.00
Stage III/IV	4/13 (30.77%)	9/13 (69.23%)	
*Tumor size (T)*			
T1–T2	10/39 (25.64%)	29/39 (74.36%)	0.55
T3	6/18 (33.33%)	12/18 (66.67%)	
*Lymph nodes metastasis (N)*			
N0	16/56 (28.57%)	40/56 (71.43%)	Count < 5
N1	0/1 (0%)	1/1 (100%)	
*Metastasis (M)*			
M0	13/50 (26.00%)	37/50 (74.00%)	0.39
M1	3/7 (42.86%)	4/7 (57.14%)

**Table 4:** Correlation analysis of ERβ expression level with patient’s age,gender,tumor stage and grade.

	ERβ expression		*P*-value (two-tailed)
	High/Middle	Low/Negative	
*Ages,year*			
≤25	8/39 (20.51%)	31/39 (79.49%)	1.00
>25	4/18 (22.22%)	14/18 (77.78%)	
*Gender*			
Male	8/37 (21.62%)	29/37 (78.38%)	1.00
Female	4/20 (20.00%)	16/20 (80.00%)	
*Tumor grade*			
Stage I/II	9/44 (20.45%)	35/44 (79.55%)	1.00
Stage III/IV	3/13 (23.08%)	10/13 (76.92%)	
*Tumor size (T)*			
T1–T2	8/39 (20.51%)	31/39 (79.49%)	1.00
T3	4/18 (44.16%)	14/18 (77.78%)	
*Lymph nodes metastasis (N)*			
N0	12/56 (17.86%)	44/56 (82.14%)	Count < 5
N1	0/1 (0%)	1/1 (100%)	
*Metastasis (M)*			
M0	12/50 (24.00%)	38/50 (76.00%)	0.32
M1	0/7 (0%)	7/7 (100%)	

### Positive correlation between the expression of BRD4 and AR in OS tissues

**Fig. 1:**
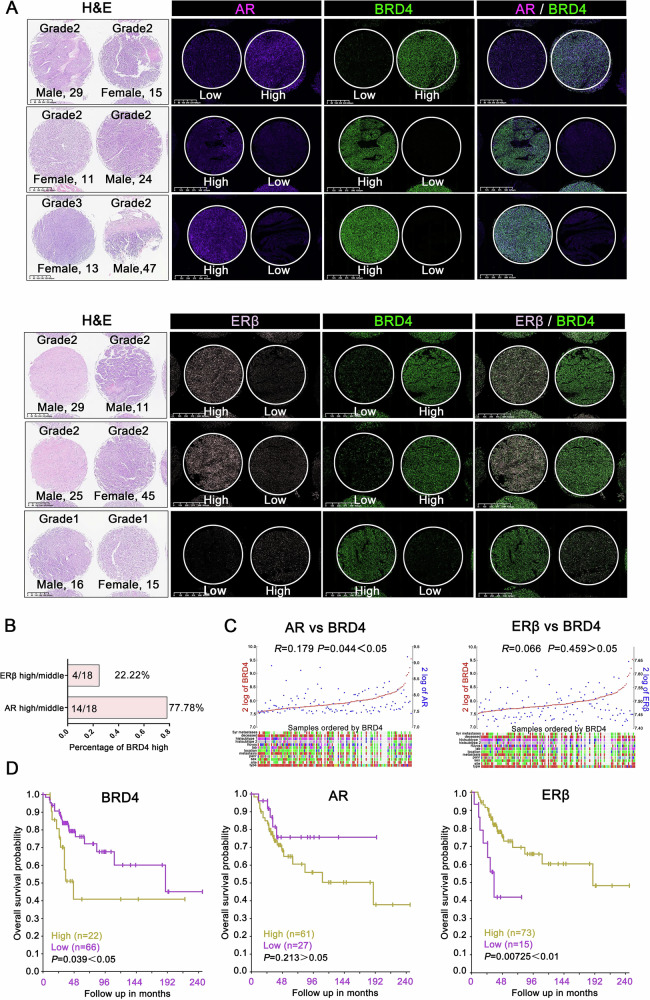
Positive correlation between BRD4 and AR expression in OS tissues. **A** Top: expression distribution of AR and BRD4; bottom: expression distribution of ERβ and BRD4. **B** Proportion of high AR and ERβ expression in 18 OS samples with elevated BRD4 levels. **C** Correlation analysis of BRD4 with AR and ERβ expression in OS tissues according to the GEO database (GSE42352), assessed using Pearson’s chi-squared test. **D** Statistical analysis of BRD4, AR, and ERβ expression levels and their correlation with OS patient survival probabilities was performed using the Kaplan-Meier analysis, based on GEO database data (GSE42352).

Based on the mIF results from TMA, a potential positive correlation was observed between AR and BRD4 expression in OS tissues. This was illustrated by selecting two adjacent tissues on the TMA slide, which showed a strong correlation in fluorescence intensity between BRD4 and AR. In contrast, the fluorescence signal for ERβ was significantly different from that of BRD4 (Fig. 1A). Quantitative analysis showed that 77.78% (14/18) of samples with high BRD4 expression also had elevated levels of AR expression, while only 22.22% (4/18) showed high expression of ERβ (Fig. 1B). This finding prompted further investigation. To expand the sample size, we included the OS gene expression microarray database (GSE42352) in our analysis. The results showed a significant positive correlation between the expression of BRD4 and AR (*P* = 0.044 < 0.05), but no significant correlation between BRD4 and ERβ (*P* = 0.459 > 0.05) (Fig. 1C). Although the public database provided transcriptional analyses, our study focus on protein expression levels. Nonetheless, integrating these data provided a more comprehensive understanding of the correlation between sex hormone receptors and BRD4 expression.

Furthermore, high BRD4 expression was significantly associated with reduced survival rates (*P* = 0.039 < 0.05). Although AR expression did not reach statistical significance (*P* = 0.213 > 0.05), a trend toward reduced survival was observed, suggesting potential clinical relevance. In contrast, increased ERβ expression was strongly associated with better prognosis in OS patients (*P* = 0.00725 < 0.01) (Fig. 1D).

This findings suggest that targeting BRD4 or androgen signaling and its receptor pathways could be a viable strategy to improve OS prognosis. However, given the substantial differences among OS subtypes, further research is needed to determine whether this strategy can be universally applied across all subtypes.

### Differential expression patterns of BRD4, AR, and ERβ across OS subtypes

**Fig. 2:**
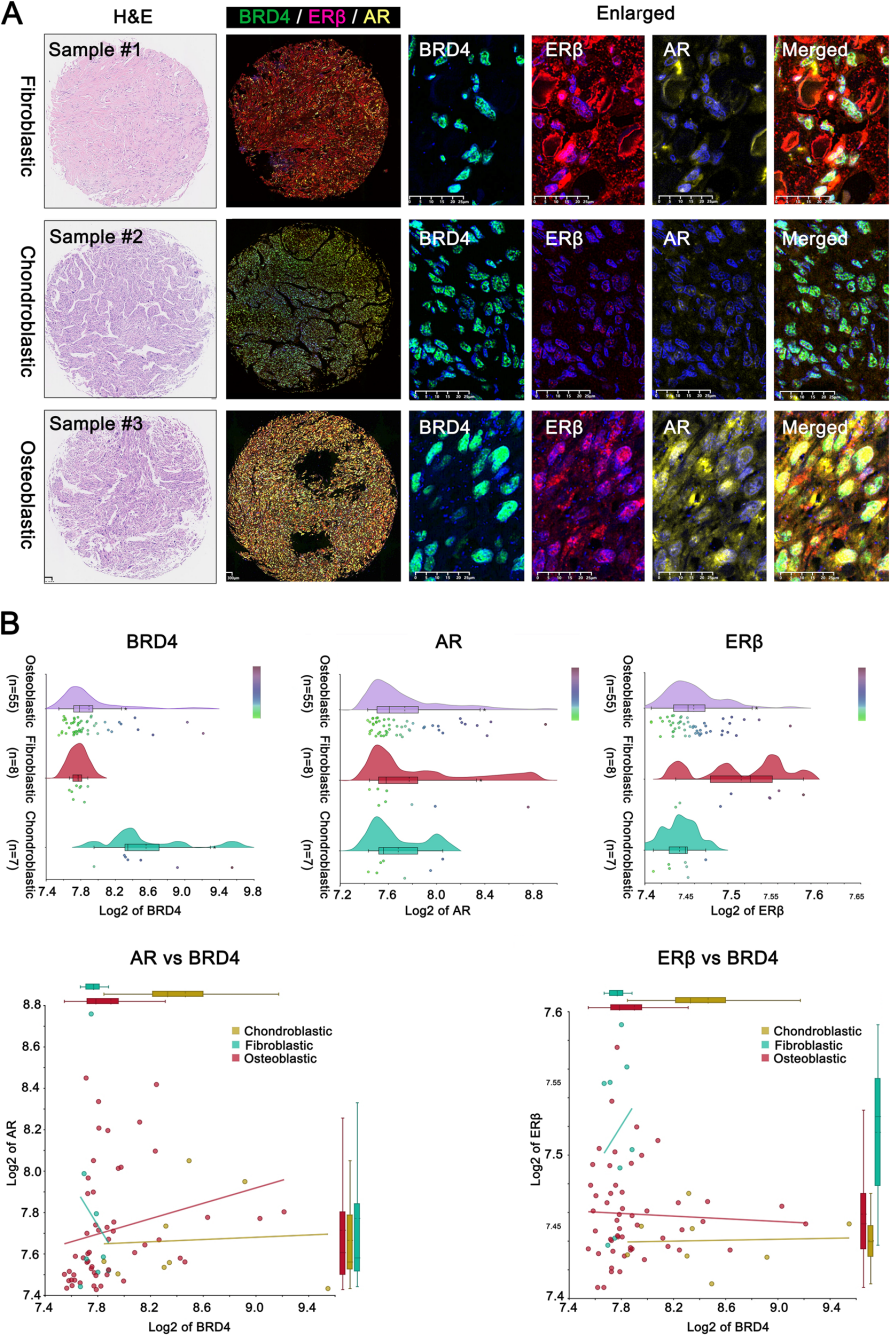
Differential expression patterns of BRD4, AR, and ERβ across OS subtypes. **A** Protein expression profiles of BRD4, AR, and ERβ in various OS subtypes (osteoblastic, fibroblastic, and chondroblastic) were illustrated with three representative tissue samples. To better distinguish the fluorescence color, we use CaseViewer software (3D-HISTECH Ltd.) to convert the original purple fluorescence signal of AR to yellow and the original pink signal of ERβ to red. **B** Transcriptional levels of BRD4, AR, and ERβ across different OS subtypes were analyzed using data from the GEO database (GSE42352).

OS originates from primitive progenitor cells with multipotent properties, enabling differentiation into osteoblasts, chondrocytes, and fibroblasts. Therefore, OS can be classified into three subtypes—osteoblastic, chondroblastic, and fibroblastic—based on the relative proportions of these cell types [16]. An intriguing observation from our investigation was that AR and ERβ exhibited distinct expression patterns across different OS subtypes (Fig. 2A). Specifically, among 20 cases of osteoblastic OS, 16 cases (80%) showed moderate to high AR expression and 6 cases(30%) exhibited moderate to high ERβ expression. Conversely, among 9 cases of fibroblastic OS, the moderate to AR expression was observed in 1 case (11.11%), while ERβ expression was moderate to high in 7 cases (77.78%) (Tables 5–7). These findings suggested that sex hormones may play roles in the progression of different OS subtypes, with AR associated with the osteoblastic subtype, which is more prevalent and characterized by high recurrence and metastasis [17]. In contrast, ERβ appears to be linked to the fibroblastic subtype, which is relatively rare and associated with a more favorable prognosis [17]. These findings may explain why high AR associated with poor prognosis in OS, while high ERβ expression may indicate a more favorable outcome. It should be noted that, due to the limited availability of only three chondroblastic OS samples, statistical analysis was not feasible, and thus this subtype was not emphasized in our discussion.

**Table 5:** Analysis of BRD4 expression level in different tumor subtypes.

	BRD4 expression		*P*-value° (two-tailed)
Osteoblastic type	12/20 (60.00%)	8/20 (40.00%)	
Fibroblastic type	3/9 (33.33%)	6/9 (66.67%)	
Chondroblastic type	1/3 (33.33%)	2/3 (66.67%)	Count < 5
Others	14/25 (56.00%)	11/25 (44.00%)

**Table 6:** Analysis of AR expression level in different tumor subtypes.

	AR expression		*P*-value° (two-tailed)
Osteoblastic type	16/20 (80.00%)	4/20 (20.00%)	
Fibroblastic type	1/9 (11.11%)	8/9 (88.89%)	
Chondroblastic type	1/3 (33.33%)	2/3 (66.67%)	Count < 5
Others	7/25 (28.00%)	18/25 (72.00%)

**Table 7:** Analysis of ERβ expression level in different tumor subtypes.

	ERβ expression		*P*-value° (two-tailed)
Osteoblastic type	16/20 (80.00%)	4/20 (20.00%)	
Fibroblastic type	1/9 (11.11%)	8/9 (88.89%)	
Chondroblastic type	1/3 (33.33%)	2/3 (66.67%)	Count < 5
Others	7/25 (28.00%)	18/25 (72.00%)	

Furthermore, data from the GSE42352 database, including 55 osteoblastic, 9 fibroblastic, and 9 chondroblastic OS cases, showed that AR correlated positively with BRD4 only in the osteoblastic subtype, while ERβ correlated positively with BRD4 only in the fibroblastic subtype. However, in the chondroblastic subtype, neither AR nor ERβ exhibited any correlation with BRD4 expression (Fig. 2B). This further highlights the importance of investigating between AR and BRD4 in the context of malignant osteoblastic OS.

### Elevated BRD4 expression in OS cells induced by DHT

**Fig. 3:**
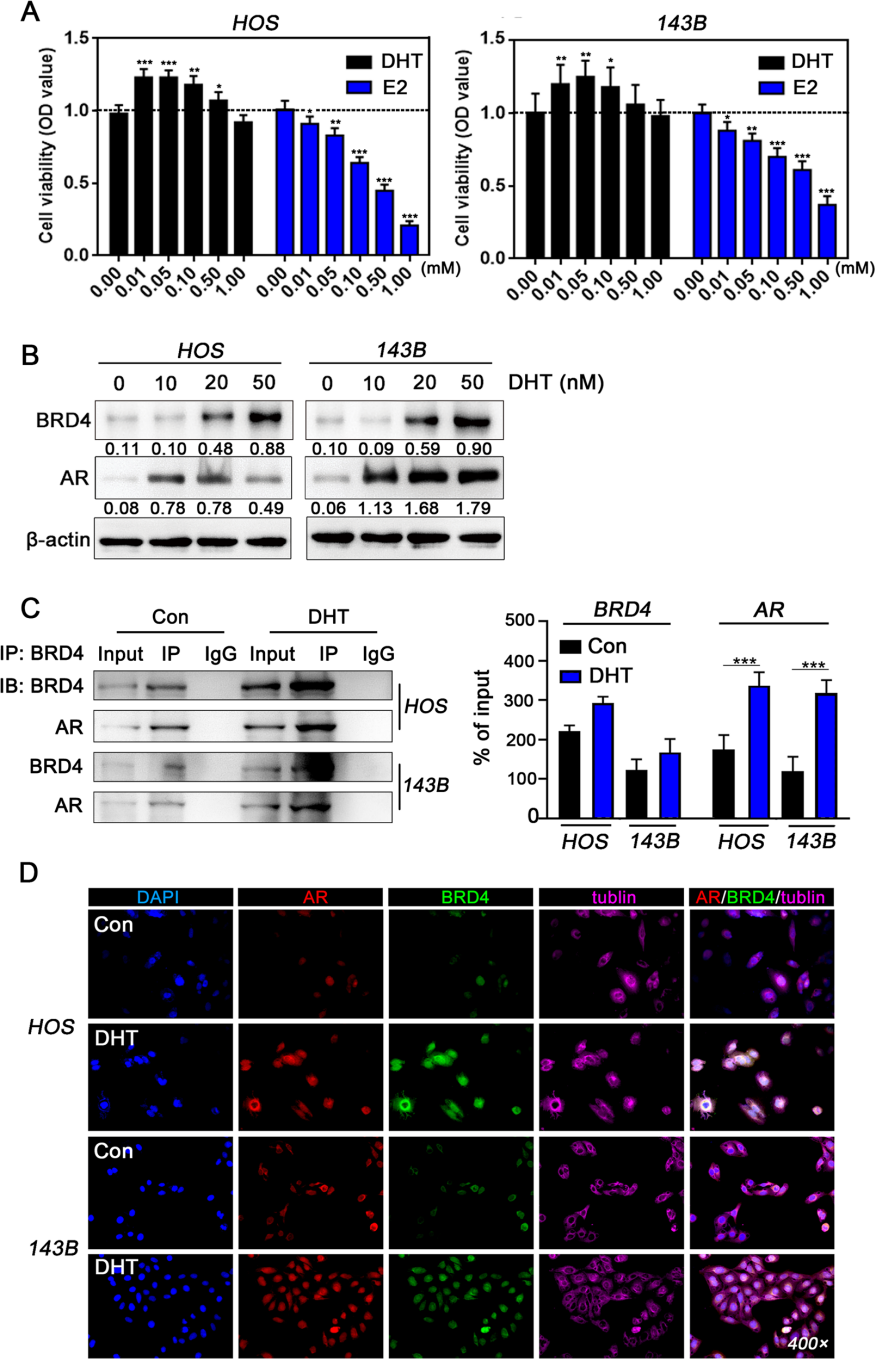
Elevated BRD4 expression in OS cells induced by androgens. **A** Cell viability of OS cells treated with varying doses of DHT (0–1 μg) or E2 (0–1 μg) was assessed via MTT assay (*n* = 6), with data analyzed using Dunnett’s test in a one-way ANOVA. **B** Western blot analysis determined the relative expression levels of BRD4 and AR proteins in OS cells stimulated with DHT at concentrations of 0, 10, 20, and 50 ng, with statistical analysis conducted using Dunnett’s test in a one-way ANOVA. **C** Co-IP assay evaluated the endogenous interaction between BRD4 and AR in OS cells treated with 20 ng of DHT for 24 h, quantifying the relative abundance of immunoprecipitated products as a percentage of input, with statistical analysis using Student’s *t*-test. **D** mIF staining was performed to observe the subcellular localization changes of AR and BRD4. *P < 0.05; **P < 0.01; ***P < 0.005.

OS cell lines HOS and 143B were treated with DHT or E2 at concentrations ranging from 0 to 1000 nM for 24 h to evaluate their effects on cell viability. The results showed that DHT significantly increased cell viability, with the maximum effect observed at 50 nM. In contrast, E2 treatment led to a concentration-dependent reduction in cell viability (Fig. 3A). Additionally, the protein levels of AR and BRD4 significantly increased under DHT treatment at concentrations ranging from 0 to 50 nM (Fig. 3B and Supplementary Fig. 2). Therefore, subsequent experiments used 20 nM DHT, which effectively increased BRD4 and AR protein expression at this lower concentration.

Since BRD4 and AR are key transcriptional regulators, we investigated the functional interactions between them in response to increased androgen signaling. Co-IP assays showed that BRD4 and AR co-immunoprecipitate in DHT-treated cells, a phenomenon absent in control samples (Fig. 3C and Supplementary Fig. 2). Subsequent mIF results revealed that, in the absence of DHT, AR primarily localized in the cytoplasm with relatively low expression levels. In contrast, DHT stimulation induced AR translocation to the nucleus, significantly increase its expression and enhancing colocalization with BRD4, as shown by overlapping fluorescence signals (Fig. 3D). These findings demonstrate the sensitivity of OS cells to androgen and suggest that androgen may promote the formation of a transcription regulatory complex involving both AR and BRD4.

### Androgens depend on BRD4 to facilitate the malignant proliferation of OS cells

**Fig. 4:**
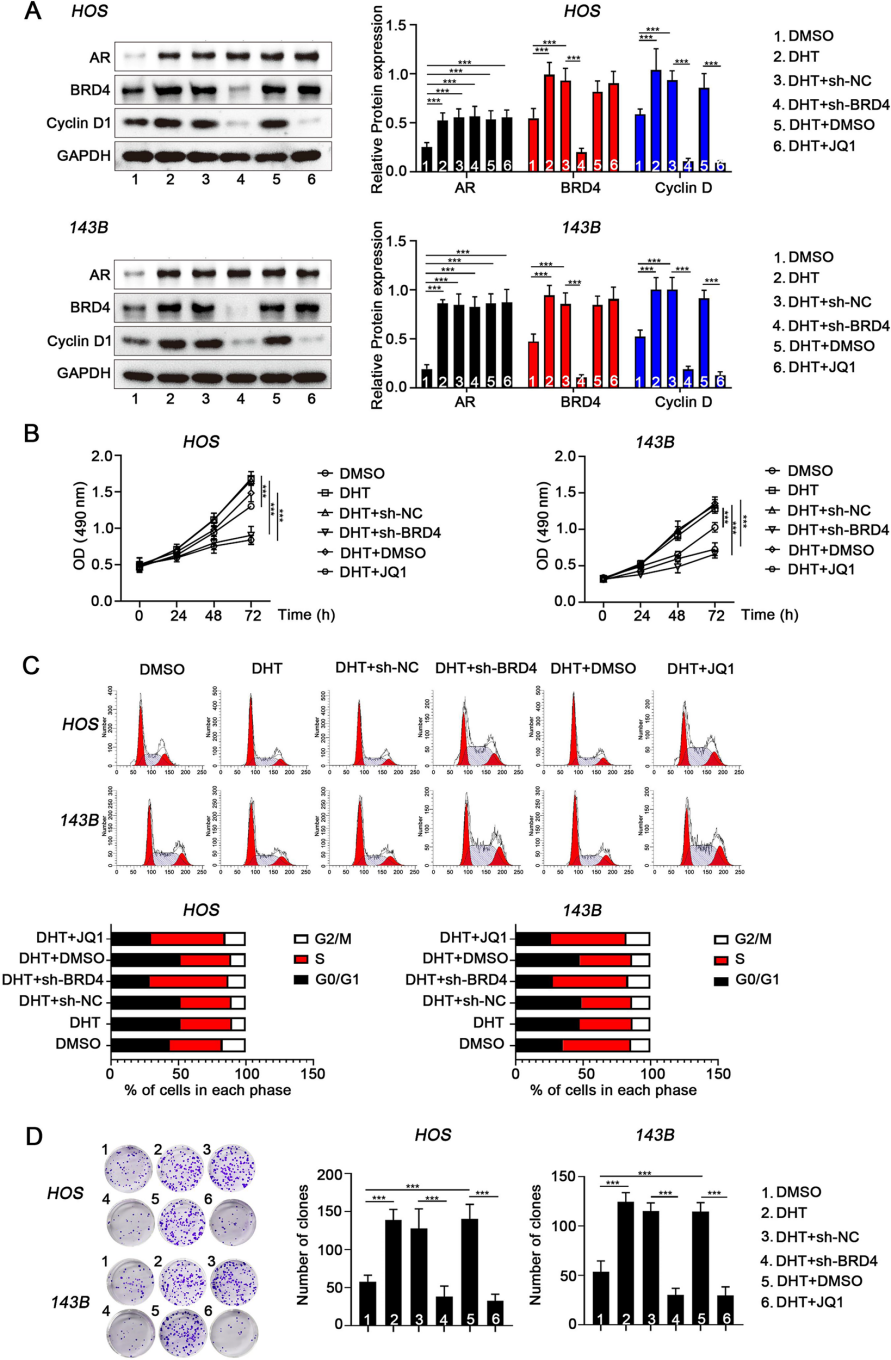
Androgens depend on BRD4 to facilitate the malignant proliferation of OS cells in vitro. **A** Western blot analysis was conducted to assess the impact of DHT and the BRD4 inhibitor (+)-JQ1 on the expression levels of AR, BRD4, and Cyclin D1 in OS cells, analyzed using Dunnett’s test following one-way ANOVA. **B** Cell viability across groups was evaluated using the MTT assay (*n* = 6), with statistical significance assessed via Dunnett’s test of one-way ANOVA. **C** Flow cytometry was employed to analyze cell cycle distribution in each group. **D** A colony formation assay was performed to determine the clonogenic potential of cells in each group, with results analyzed using Dunnett’s test of one-way ANOVA. *P < 0.05; **P < 0.01; ***P < 0.005.

A cellular function-loss model of BRD4, using siRNA and (+)-JQ1, was employed to investigate BRD4’s role in mediating androgen effects on the malignant proliferation of OS cells. Our findings showed that DHT significantly enhanced cell proliferation, as indicated by accelerated cell cycle progression, increased clonogenicity, and elevated Cyclin D1 protein levels. In contrast, BRD4 inhibition significantly reduced the proliferative capacity of OS cells (Fig. 4A–D and Supplementary Fig. 3).

**Fig. 5:**
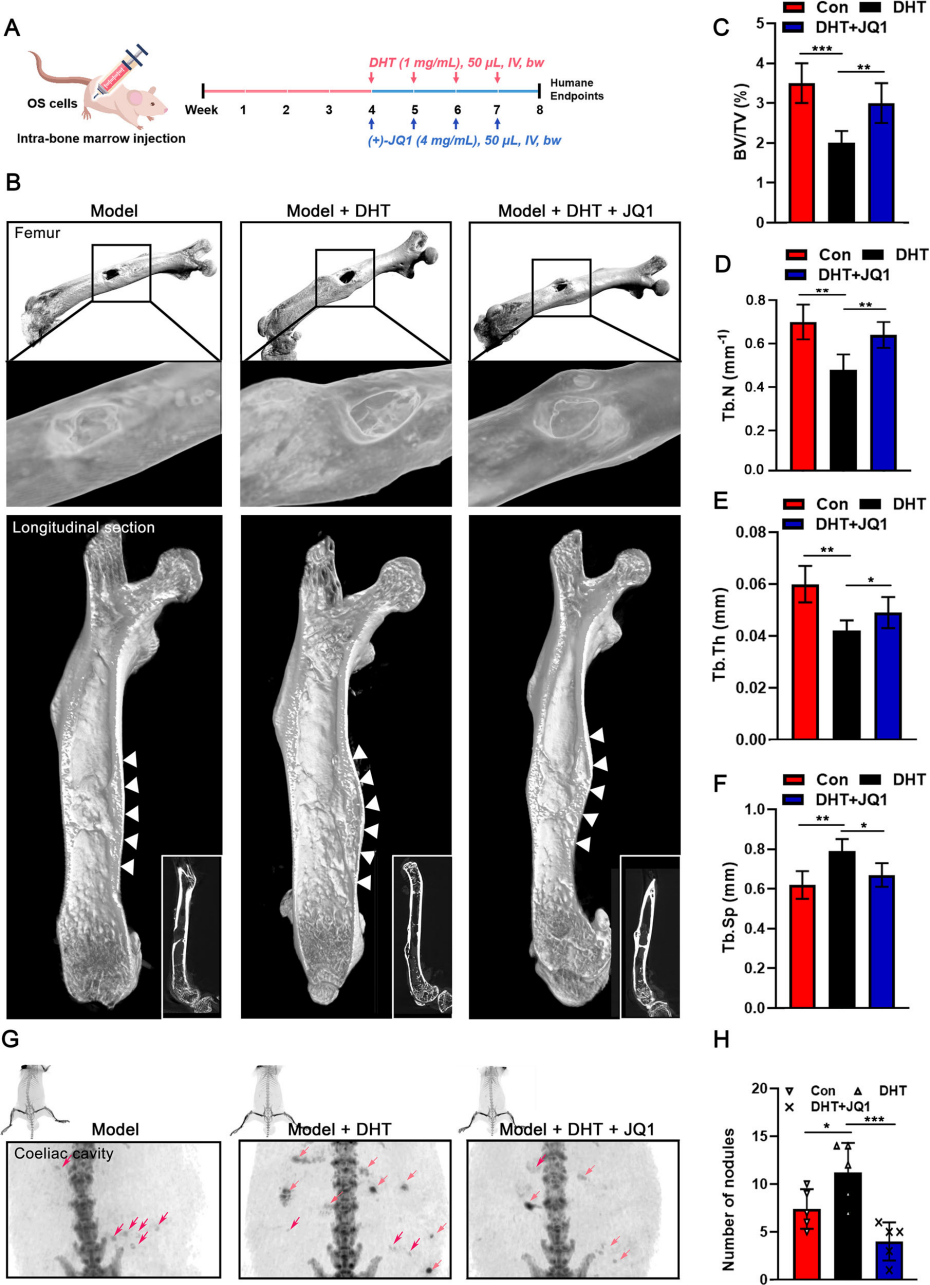
Androgens depend on BRD4 to facilitate the malignant proliferation of OS cells in vivo*.* **A** Flowchart illustrating the animal experimental procedure. **B** Micro-CT imaging and 3D reconstruction results of mouse femur tissues. **C** Ratio of BV/TV. **D** Trabecular bone number (Tb.N). **E** Trabecular bone thickness (Tb.Th). **F** Trabecular separation (Tb.Sp). **G** Micro-CT findings of lymph nodes in coeliac cavity (intestinal). **H** Quantification of lymph nodules in each group of mice. In (**C**−**F**) and (**H**), ANOVA. *P < 0.05; **P < 0.01; ***P < 0.005.

**Fig. 6:**
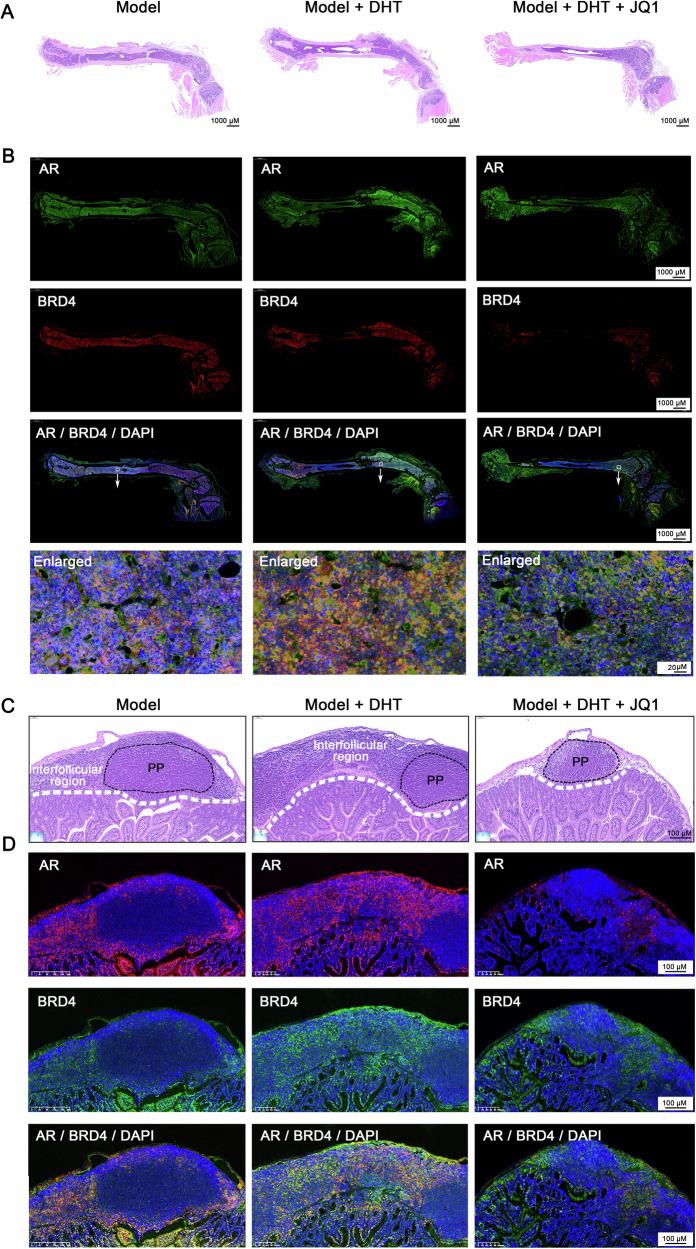
Androgens depend on BRD4 to facilitate the malignant proliferation of OS cells in vivo*.* Histopathological examination of femur (**A**) and intestinal lymph nodules (**B**) via H&E staining. Expression levels of AR and BRD4 in femur (**C**) and intestinal lymph nodules (**D**) detected by the mIF technique, *n* = 5.

Next, an orthotopic xenograft model of HOS cells in the bone marrow cavity of nude mice was established to evaluate the effects of androgens on OS progression and dependence on BRD4 in vivo (Fig. 5A). Micro-CT imaging showed that the femoral diaphysis surface in DHT-treated mice was significantly rougher compared to the untreated group. Additionally, a distinct bony bulge near the tumor injection site was observed, along with signs of bone destruction (Fig. 5B). Furthermore, the DHT-treated mice showed significant reductions in femoral bone density (BV/TV), trabecular number (Tb.N), and trabecular thickness (Tb.Th), as well as increased in trabecular separation (Tb.Sp). These findings suggest that elevated DHT levels can cause more severe disruption of bone structure by tumor cells, leading to pronounced osteoporotic changes (Fig. 5C–F). In contrast, (+)-JQ1 treatment mitigated the effects of DHT, reducing bone morphological destruction and bone loss (Fig. 5B–F). Notably, the mouse model established using this method showed significant enlargement of the intestinal Peyer’s patches (PP), potentially linked to cancer cell metastasis. Importantly, (+)-JQ1 treatment significantly reducing lymph node enlargement (Fig. 5G and H), suggesting the therapeutic potential of targeting BRD4 to address OS metastasis. Moreover, mIF analyses showed elevated expression of AR and BRD4 in the bone marrow and the interfollicular region of PP in DHT-treated mice. However, (+)-JQ1 administration significantly counteracted effects of DHT, leading to a marked reduction in BRD4 expression in these tissues (Fig. 6A–D). These findings highlight that DHT’s promotion of OS cell proliferation depends on BRD4 functionality both in vitro and in vivo settings.

### Androgen-induced BRD4 expands chromatin distribution and exhibits high affinity with AR

**Fig. 7:**
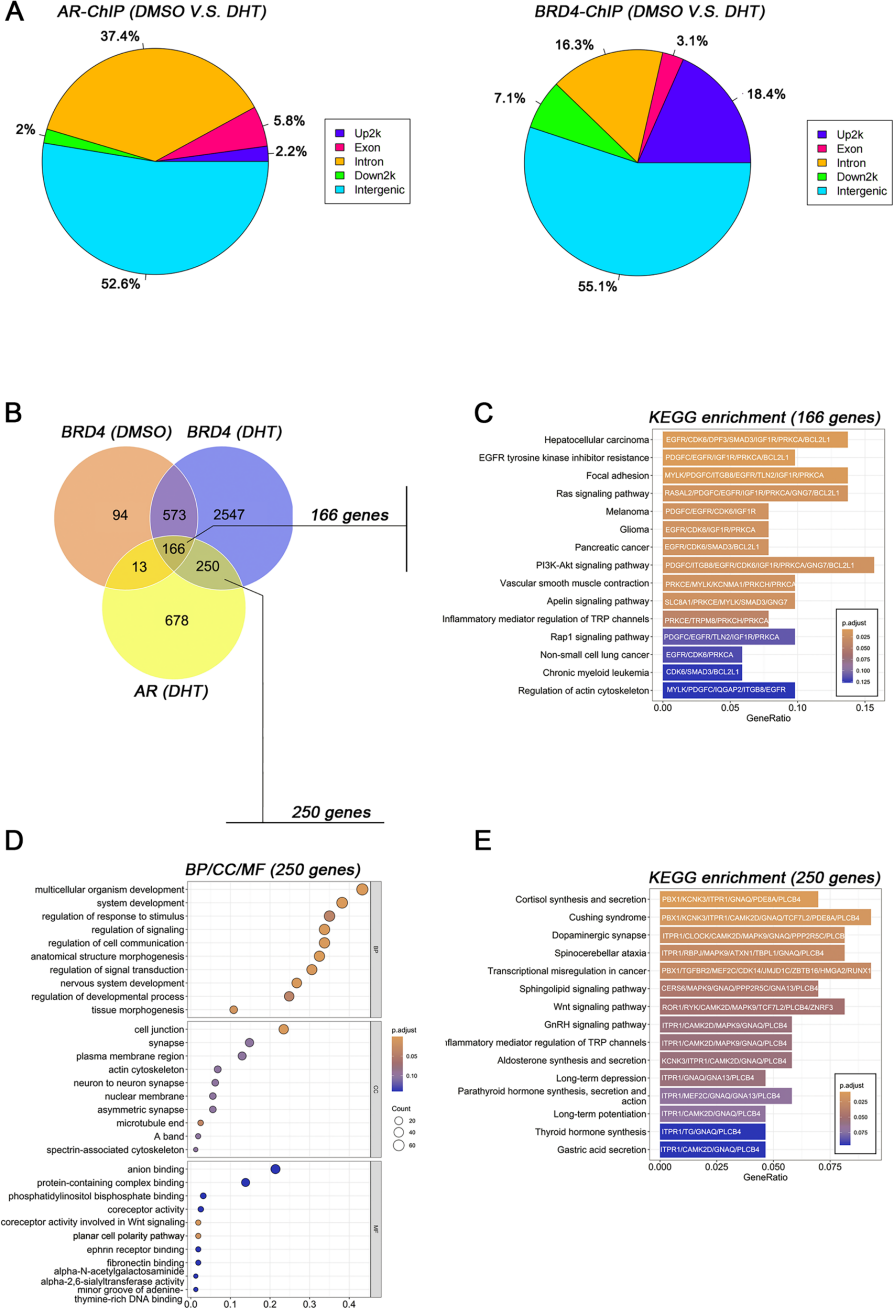
Androgen-induced BRD4 expands chromatin distribution and exhibits high affinity with AR. **A** Pie chart illustrating the changes in the chromatin distribution of AR and BRD4 before and after DHT stimulation. **B** Venn diagram depicting the relationship between the total number of genes regulated by BRD4 and AR before and after DHT induction. **C** KEGG pathway enrichment analysis of the 166 genes potentially co-regulated by BRD4 and AR, highlighting the top 15 signaling pathways. **D** Gene Ontology (GO) analysis of the 250 genes co-regulated by BRD4 and AR exclusively after DHT induction. **E** KEGG pathway enrichment analysis for the signaling pathways associated with the genes in (**D**), showcasing the top 15 pathways.

We performed ChIP-Sequencing analysis of BRD4 and AR to investigate their chromatin distribution after DHT stimulation. The results revealed that DHT treatment significantly altered the intergenic distribution of both BRD4 and AR, with over half of the signals showing changes (AR: 52.6%; BRD4: 55.1%). This suggests that androgen can alter the transcriptional programs regulated by AR and BRD4 (Fig. 7A). Additionally, DHT induction expanded BRD4’s chromatin distribution, increasing the total number of associated genes from 846 to 3536, a 4.2-fold increase. Notably, 2797 of these BRD4-related genes were newly identified following DHT treatment, account for 79.1% of all involved genes (Fig. 7B). See Supplementary File for complete ChIP-seq datasets documenting BRD4 and AR occupancy on chromatin.

Further analysis showed that the chromatin localization of BRD4 and AR became more convergent after DHT stimulation, with the overlap ratio increasing from 16.16% (179/1107) to 37.57% (416/1107). We identified 166 conserved genes co-regulated by BRD4 and AR, regardless of DHT stimulation. These include key genes involved in various signaling pathways, such as platelet-derived growth factor C (PDGFC), insulin-like growth factor 1 receptor (IGFR1), epidermal growth factor receptor (EGFR), B-cell lymphoma 2-like 1 (BCL2L1), and cyclin-dependent kinase 6 (CDK6), which are primarily enriched in PI3K/AKT-related cell proliferation and anti-apoptotic pathways. In contrast, 250 genes were newly identified as co-regulated by AR and BRD4 only after DHT induction, including phospholipase Cβ4 (PLCB4), guanine nucleotide-binding protein G(q) subunit alpha (GNAQ), and inositol 1,4,5-trisphosphate receptor type 1 (ITPR1), which are primarily involved in lipid metabolism and corticosterone production and secretion (Fig. 7C–E). These findings suggest that DHT significantly influences the transcriptional regulatory programs of BRD4 and AR, potentially driving increased proliferation and metabolic activity.

### Enhanced expression of PLCB4 by formation of AR-BRD4 transcriptional regulatory complex

**Fig. 8:**
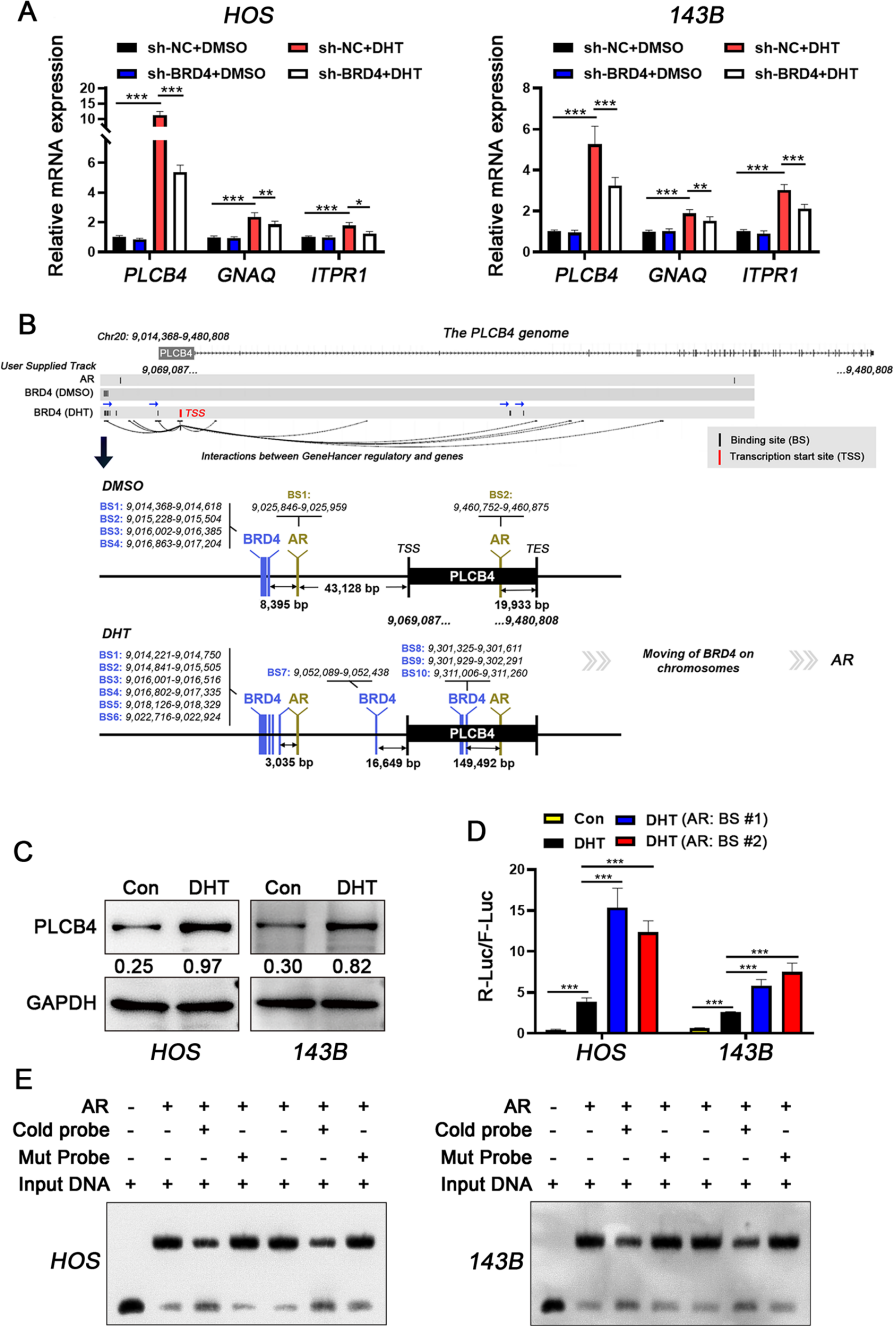
Enhanced expression of PLCB4 by formation of AR-BRD4 transcriptional regulatory complex. **A** RT-qPCR analysis of the relative mRNA expression levels of PLCB4, GNAQ, and ITPR1 in various groups. **B** Schematic representation of the spatial distribution of AR and BRD4 binding sites on the PLCB4 genome. **C** Western blot analysis of the relative protein expression level of PLCB4 following DHT induction. **D** Dual-luciferase assay evaluating the impact of each AR binding sites on the activity of the PLCB4 promoter. **E** The EMSA technique was employed to confirm the binding capability of the AR-BS#1 probe with AR. Statistical significance was determined using Dunnett’s test for one-way ANOVA. *P < 0.05; **P < 0.01; ***P < 0.005.

The focus of our investigation was to identify target genes co-regulated by BRD4 and AR in response to DHT. To this end, we assessed the mRNA expression levels of PLCB4, GNAQ, and ITPR1 in OS cells before and after DHT treatment. The results showed a significant increase in the relative mRNA levels of these three genes following DHT stimulation, especially in cells with unaltered BRD4 expression (Fig. 8A). Therefore, we focused on the PLCB4 gene in subsequent studies due to its high sensitivity to DHT in transcriptional activity.

**Fig. 9:**
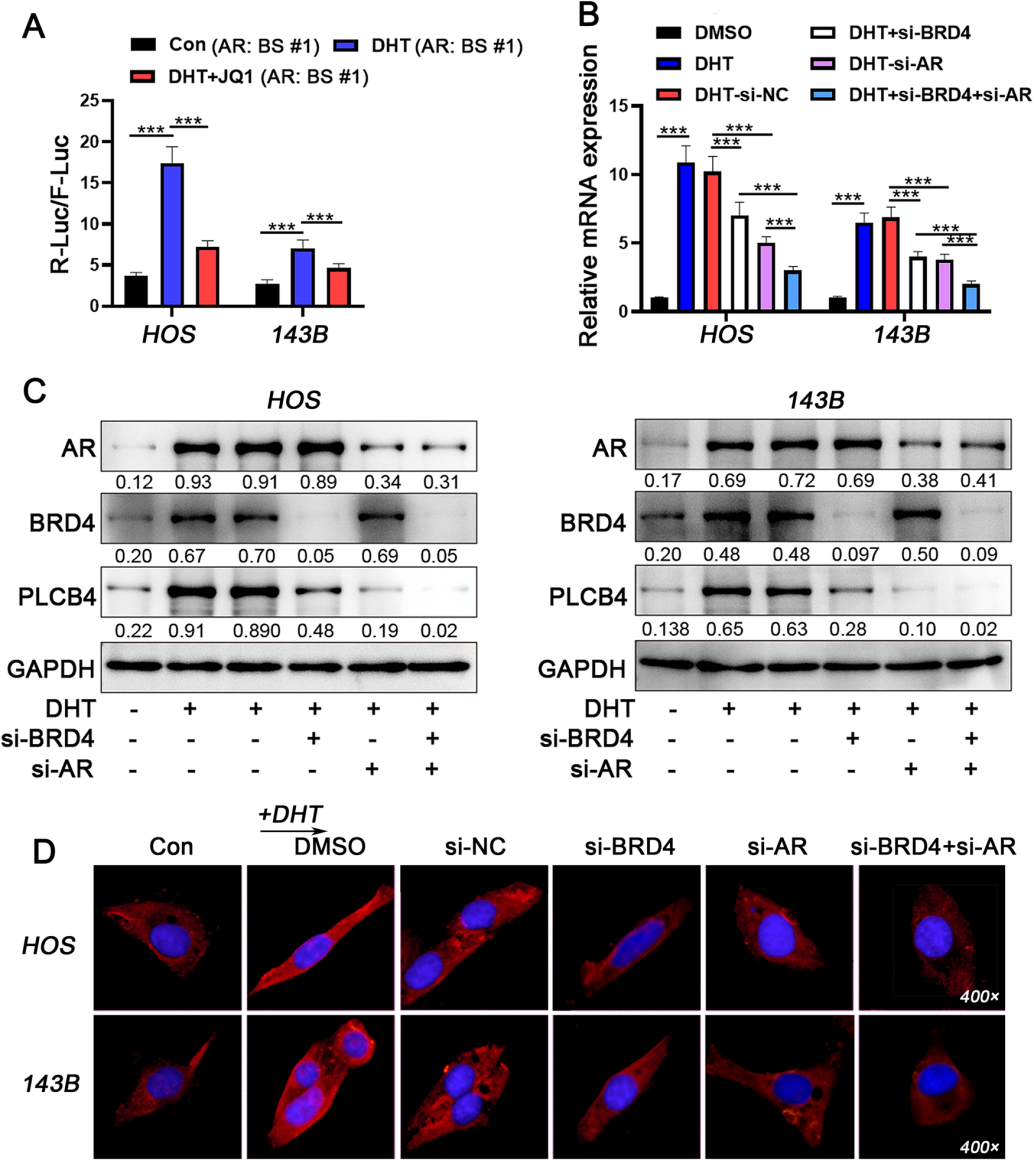
Enhanced expression of PLCB4 by formation of AR-BRD4 transcriptional regulatory complex. **A** The impact of individual and combined inhibition of AR and BRD4 on PLCB4 mRNA and protein expression levels was assessed using RT-qPCR (**B**) and western blot analysis (**C**). **D** mIF analysis was performed to evaluate the expression levels of PLCB4 in each groups. Statistical significance was determined using Dunnett’s test for one-way ANOVA. *P < 0.05; **P < 0.01; ***P < 0.005.

Using the *UCSC Genome Browser’s Custom Tracks* for chromatin location analysis, we identified two AR binding sites in the PLCB4 genomic region: BS #1, located 43,128 bp upstream of the transcription start site (TSS), and BS #2, 19,933 bp upstream of the transcription end site (TES). Additionally, several BRD4 binding sites were identified upstream of the TSS, initially located at least 8395 bp from the AR-BS #1 in control cells. However, this distance decreased to 3035 bp in DHT-treated cells (Fig. 8B), indicating that DHT not only increased the number of BRD4 binding sites on the PLCB4 genome from 4 to 10 but also brought these sites a closer to the AR binding sites, significantly elevating PLCB4 expression in the presence of DHT (Fig. 8C and Supplementary Fig. 4). Further analysis of the transcriptional regulatory roles of the two AR binding sites showed that the BS #1 significantly enhanced PLCB4 promoter activation in response to DHT (Fig. 8D). Additionally, EMSA assay confirmed the interaction between this binding site and AR in OS cells under the treatment of DHT (Fig. 8E and Supplementary Fig. 4). However, dual luciferase reporter assays showed that, even with the BS #1 present, (+)-JQ1 significantly reduced DHT-stimulated transcriptional activity, indicating the dependence of this transcriptional regulatory program on BRD4 (Fig. 9A). Furthermore, knocking of either AR or BRD4 reduced PLCB4 transcriptional levels, with the most significant reduction observed when both AR and BRD4 were knocked down simultaneously (Fig. 9B–D and Supplementary Fig. 5). These present data demonstrate that DHT enhances the formation of the AR-BRD4 transcriptional regulatory complex, leading to the upregulation of PLCB4 in OS cells.

### The carcinogenic roles of PLCB4 in OS

**Fig. 10:**
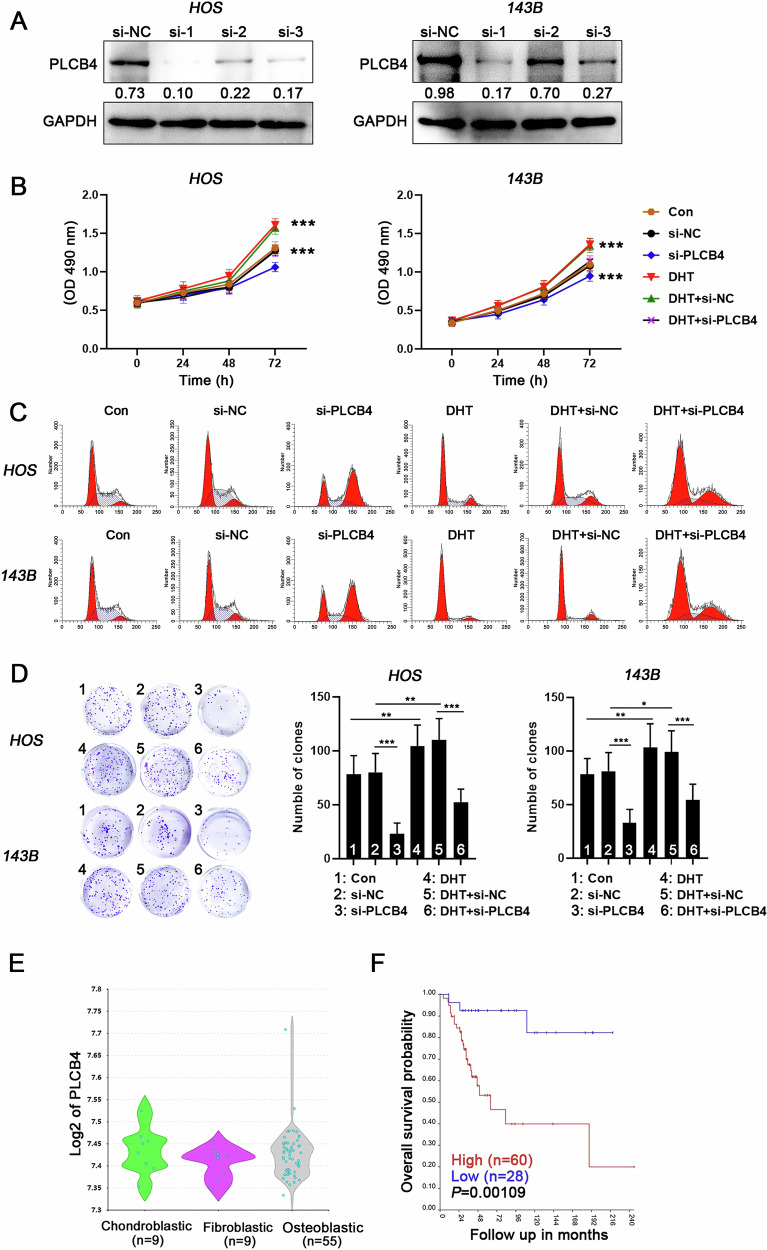
The carcinogenic roles of PLCB4 in OS. **A** Western blot analysis of PLCB4 protein expression across different groups. **B** MTT assay measuring cell viability (*n* = 6). **C** Flow cytometry analysis of cell cycle. **D** Clonogenic assays evaluating the proliferative capacity of cells. ANOVA, *P < 0.05; **P < 0.01; ***P < 0.005. **E** Examination of PLCB4 transcription levels among different OS subtypes utilizing the GEO database (GSE42352). **F** Kaplan–Meier analysis linking PLCB4 transcription levels with survival outcomes in OS patients.

Since the role of PLCB4 in OS cells has not been studied, we used siRNA technology to inhibit PLCB4 expression and investigate its impact on OS cells proliferation (Fig. 10A and Supplementary Fig. 6). Our results showed that reducing PLCB4 levels significantly decreased cell viability, disrupted the cell cycle, and impaired the clonogenic ability of DHT-stimulated OS cells (Fig. 10B–D). Moreover, analysis of GSE42352 database revealed that PLCB4 expression is higher expression in osteoblastic and chondroblastic OS subtypes compared to the fibroblastic subtype (Fig. 10E), This trend resembles the expression patterns of AR and BRD4 across different subtypes. Furthermore, Kaplan-Meier analysis of OS patients showed that high PLCB4 expression was significantly associated with reduced survival probability (*P* = 0.001 < 0.01) (Fig. 10F). These findings further suggests that PLCB4 is a promising therapeutic target in OS.

## Discussion

Skeletal development is primarily regulated by multi-hormone, including pituitary growth hormone, thyroid hormone, androgen and estrogens. During this stage, the pattern in which girls grow faster than boys in preadolescent period changes, and by the mid-to-late adolescence, males become considerably taller [18, 19]. This coincides with the epidemiological features of OS, which has its first peak incidence at puberty and a higher incidence in males than in females’ [2].

Evidence for specific markers in pathological tissues comes from studies using various techniques. In pathological examination, immunohistochemistry is the most commonly used technique in these studies [20–25]. For example, Osamu et al. were the first to systematically examined sex steroid receptors in OS patient samples [24]. However, the sample sizes was relatively small. In this study, we examined AR, ERa, ERβ, and aromatase expression in 57 analyzable OS tissue samples, supplementing previous studies on OS hormone etiology and enabling more accurate analysis.

Sex steroids, such as estrogen and androgen, not only regulate osteocyte proliferation and apoptosis [26, 27], but may also directly affect pluripotent mesenchymal progenitor cells, which were the hypothesized origin cells of OS [28]. Although the role of sex steroids in OS has rarely reported, Scranton Jr. et al. demonstrated that estrogen significantly inhibits proliferation in primary OS cells isolated from patients [29]. Similarly, interference also inhibited OS cell proliferation, consistent with the effects of estrogen [30, 31]. In this study, DHT and E2 exhibited opposite effects in OS cells in vitro. Specifically, DHT induced high expression of AR and BRD4 and enhanced their endogenous interaction. However, E2 intervention significantly downregulated OS cell proliferation, consistent with the anti-tumor effect of ERβ reported by Yang et al. [32] in 143B cells. Thus, the results suggest that androgen promote malignant proliferation of OS, whereas estrogen has the opposite effect.

However, although androgen is essential for bone development, it is not the sole inducer of OS. The ‘single driver’ hypothesis proposes that each OS subtype is initiated by a subtype-specific driver, such as a genetic mutation, in proliferative cells [33]. In a study involving exome sequencing of 1004 OS patients, pathogenic or likely pathogenic cancer-susceptibility gene variant was identified in 281 patients (28.0%), with nearly three-quarters of these variant linked to autosomal-dominant genes or known OS-associated cancer predisposition syndrome genes [34]. These findings suggested that genetic mutations likely played significant roles in OS development. However, our study demonstrated that OS cells were highly sensitive to androgen, leading to increase proliferation. Therefore, elevated androgen levels may facilitate the transmission and accumulation of pathogenic genetic mutations in cells, potentially explaining the higher incidence of OS in males compared to females.

It was also worth noting that aromatase, a rate-limiting enzyme responsible for the endogenous conversion of androgens into estrogen [26], was almost absent in OS tissues. Survival analysis revealed that OS patients with higher aromatase expression had significantly shorter survival rates (*P* = 0.039 < 0.05) (Supplementary Fig. 7), suggesting that dysregulated endogenous hormones may contribute to the poor prognosis of OS. Additionally, although androgen significantly induced BRD4 expression, negatively impacting OS prognosis, the direct relationship between androgen and prognosis requires further investigation, as no statistically significant correlation was found between high AR expression levels and lower survival rates.

BRD4, functioning as a cofactor, interacts with the mediator complex co-localized at super-enhancer (super-ENHs-SE) to regulate gene expression and promote transcription elongation [7]. AR, a receptor that binds to androgen, can bind to DNA sequences known as androgen response elements (AREs), altering chromatin conformation and promote gene transcription [35, 36]. In prostate cancer, AR activates enhancers and alters target gene expression by forming complexes with co-regulatory proteins, such as the chromatin remodeling protein p300/CBP22 [20, 37], which, like BRD4, is a major component of super-enhancer (super-ENHs-SE). In this study, we found that DHT increased BRD4 expression and its binding with AR in OS cells. This suggests a potential synergistic mechanism between BRD4 and AR, acting as a super-enhancer under androgen stimulation. Consequently, this interaction likely enhanced the transcription of target genes, such as PLCB4, and promoted the OS cells proliferation.

Previously, melanoma was the only cancer associated with abnormal PLCB4 expression, driven by androgens [38, 39]. However, in OS research, only one study has identified PLCB4, along with eight other genes, as a reliable predictor of poor prognosis in OS [40]. This study provides experimental evidence supporting PLCB4 as an androgen-dependent gene involved in the malignant proliferation of OS. Specifically,we found that BRD4 typically localizes to distal regulatory regions of the PLCB4, far from the transcription start site (TSS). However, DHT treatment led to a more extensive distribution of BRD4 within these regulatory regions, with a tendency to migrate closer to the AR binding sites. Notably, BRD4-AR complexes were detected in DHT-treated cells but not in untreated cells. This suggests that DHT induces the formation of AR-BRD4 regulatory complexes, activating PLCB4 transcription. As a gene regulated by both AR and BRD4, PLCB4 significantly enhances OS cell proliferation. Moreover, the strong association between high PLCB4 expression and poor survival rates suggested that elevated PLCB4 levels may be significant risk factor for OS patients.

**Fig. 11:**
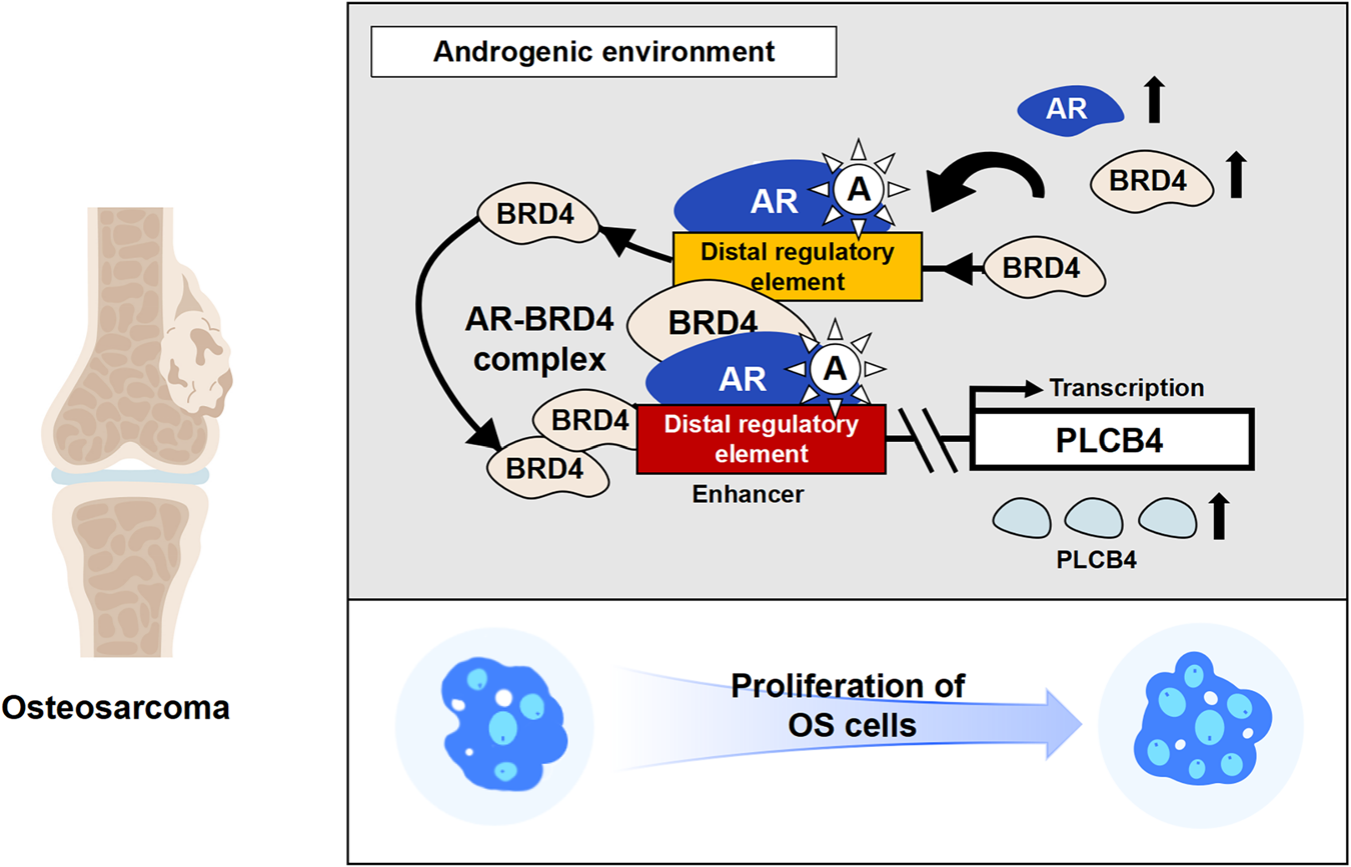
Mechanistic schematic of this study. Androgen induces the assembly of its receptor AR with BRD4 into a transcriptional regulatory complex, which activates PLCβ4 transcription and drives malignant proliferation of osteosarcoma cells.

In conclusion, our findings demonstrate that the AR-BRD4 transcriptional complex orchestrates PLCβ4 activation, thereby establishing a direct mechanistic link between androgen-driven signaling and the malignant proliferation of OS cells (Fig. 11).

## Materials and methods

### H&E staining

The OS tissue microarray (TMA) sections were purchased from AiFang Biotechnology Co., Ltd. (Changsha, Hunan, China). Sections (5 μM) were heated at 60 °C for 20 min for dewaxing, in preparation for subsequent H&E staining procedures. Specifically, hematoxylin solution was applied for 10 min to stain the nuclei, and the slips were then rinsed and differentiated with acid alcohol. Next, eosin solution was applied for 2 min to stain cytoplasm. After rinsing, the slips was dehydrated, cleared, and mounted. Stained slides were scanned through the pathological section scanner (3D-HISTECH, Cat. No. P250 FLASH) for histopathological analysis.

### Antibodies and multi-immunofluorescence (mIF) staining

The tyramide signal amplification (TSA) fluorescence system kit (RS0038; Immunoway, Suzhou, China) was employed to perform multiplex immunofluorescence (mIF) staining according to the manufacturer’s protocol. Briefly, OS TMA sections were sequentially processed as follows: fixation with 4% paraformaldehyde (PFA), permeabilization with 0.1% Triton X-100, and blocking with 1% bovine serum albumin (BSA). Following antigen retrieval, sections were incubated with a primary anti-BRD4 antibody (diluted 1:1000; Abcam, Cambridge, MA, USA) at 37 °C for 1 h. This was followed by incubation with HRP-conjugated secondary antibody (diluted 1:10,000, Abcam) in the dark for an additional hour. Subsequently, the ‘color development’ component from the TSA kit was added to enable fluorescence presentation. In this step, HRP catalyzed the addition of a tyramide fluorescent substrate to the reaction system, resulting in the generation of activated fluorescent substrate that covalently binds to tyrosine residues or other residues on the antigen. This leads to stable covalent binding of the tyramide fluorescent substrate on the sample. The antigen retrieval process was repeated, and anti-AR antibody (diluted 1:1000, Abcam) along with HRP-linked secondary antibody (diluted 1:10,000, Abcam) and the ‘color development’ component from the kit were added. This procedure was replicated until ERα (diluted 1:1000, Abcam), ERβ (diluted 1:10,000, Abcam), and aromatase (diluted 1:1000, Abcam) were each labeled with distinct fluorescent colors on the slip. Finally, the slip was cleaned with PBS to eliminate residual fluid and stained with DAPI for 10 min at 25 °C. The fluorescence excitation wavelengths for the various colors were as follows: blue (nucleus, DAPI, EX: 480 nm), green (BRD4, FITC, EX: 520 nm), orange (ERα, Rhodamine Red-X, EX: 570 nm), pink (ERβ, Cy5, EX: 590 nm), purple (AR, Cy7, EX: 780 nm), and red (aromatase, Texas Red, EX: 620 nm). Images of the specimens were captured using a KFBio slide viewer (KFBio, Ningbo, Hangzhou, China). In total, 57 valid patients’ OS tissues on the TMA slip were obtained for analysis.

### Cell culture, treatment, and transfection

OS cell lines HOS and 143B, authenticated by short tandem repeat (STR) profiling and confirmed mycoplasma-free, were acquired from Fenghui Biotechnology Co., Ltd. (Changsha, Hunan, China). Cells were maintained in high glucose DMEM (Thermo Fisher Scientific, MA, US) supplemented with 10% fetal bovine serum (FBS) (Invitrogen, Carlsbad, CA, USA) at 37 °C, 5% CO_2_ in humidified air. For transfection or cell cycle synchronization, cells in each group were serum-starved in 0.5% FBS for 12 h. DHT and E2 (Merck, Darmstadt, Germany) were treated at concentrations of 0, 10, 50, 100, 500, and 1000 nM for 24 h in OS cell lines to observe their effects on cell viability. Subsequently, 20 nM DHT or 10 μM (+)-JQ1 (Selleck CN, Shanghai, China) was treated with OS cells to observe the impact on their proliferation capacity. In gene expression interference experiments, the cells were initially seeded in a six-well plate and incubated for 24 h. The siRNAs (Thermo Fisher Scientific) for BRD4, AR, or PLCB4 was then mixed with Lipofectamine 3000 (Invitrogen) and introduced into the culture medium for 48 h.

### Detection of relative mRNA levels of specific genes via real-time fluorescent quantitative PCR (RT-qPCR)

Total RNA was isolated from OS cell lines using the TRIzol reagent (Vazyme, Nanjing, China). Subsequently, 500 ng of each RNA sample was used for cDNA synthesis with a reverse transcription kit (Promega, Madison, WI, USA). Subsequent qPCR analysis was conducted using SYBR green-based protocols with GAPDH serving as the internal control for normalization. The 2^−ΔΔCT^ method was used for quantification of target genes’ expression levels. Primers for RT-qPCR were obtained from GeneScript (Nanjing, Jiangsu, China).

### Detection of relative expression levels of specific proteins via western blotting

Total protein lysates from OS cells lines were separated by sodium dodecyl sulfate–polyacrylamide gel electrophoresis and transferred onto a PVDF membrane (Millipore, MA, USA). Then, primary antibodies of BRD4 (1:500 dilution, Immunoway), AR (1:500 dilution, Abcam), and PLCB4 (1:500 dilution, Immunoway) were added to the PVDF membranes, and incubated overnight at 4 °C. Subsequently, the corresponding horseradish peroxidase (HRP)-conjugated secondary antibodies (1:3000 dilution, Abcam) were incubated at 25 °C for another 1 h, after which signals were detected by chemiluminescence using a chemidoc system (Bio-Rad, Munich, Germany). Data were finally analyzed using Image J software V1.8.0 (National Institutes of Health, MD, USA).

### Detection of the interaction between BRD4 and AR by co-immunoprecipitation (co-IP) assay

The co-IP assay was employed to investigate the interaction between AR and BRD4. Initially, HOS and 143B cells were treated with or without 20 nM DHT for 24 h. Following treatment, protein extracts were prepared from the cells for subsequent analysis. A IP grade BRD4-specific antibody (1:100 dilution, Abcam) was added to the protein extracts and incubated overnight at 4 °C to facilitate antibody–antigen binding. Protein A/G magnetic beads (MedChemExpress, Shanghai, China) were then introduced to the mixture and allowed to incubate for an additional period to promote the formation of immune complexes. Subsequently, the beads were pelleted and subjected to multiple washes to eliminate any non-specific interactions. Finally, the immune complexes were eluted from the beads and analyzed via western blotting to detect the presence of AR protein.

### Detection of cell proliferation activity via MTT

HOS or 143B cells with function-gain or function-loss of each target gene were plated into 96-well plates. Afterward, cells were treated with different concentrations of DHT, E2, or the BRD4 inhibitor (+)-JQ1 and incubated for 24 h. Cell viability was determined using an MTT assay kit following the manufacturer’s instructions (Merck). The absorbance at 450 nm was measured using a microplate reader (Bio-Rad Laboratories).

### Detection of cell phases via flow cytometry

HOS or 143B cells from each group were collected and fixed in 70% ice-cold ethanol overnight. Subsequently, the cells were rinsed with PBS and subjected to staining with propidium iodide (PI) solution supplemented with RNase A. The stained cells were then assessed utilizing flow cytometry (Beckman, CytoFLEX, CA, USA) to measure the DNA content, and the various cell cycle phases, including G0/G1, S, and G2/M, were identified based on the DNA content. Data were analyzed to determine cell cycle phase distribution.

### Detection of clonal formation ability

Cellular proliferative capacity of each groups was investigated using a flat clone detection assay. Firstly, cells were pre-treated according to study needs and subsequently seeded at a density of 500 cells per well in a six-well plate and allowed to attach overnight. Subsequently, the cells were treated with a proliferation-inducing agent for a specific period of time. After 2 weeks of observation, the cells were fixed and stained with crystal violet (Solarbio, Beijing, China). The number of flat clones, defined as a cluster of more than 50 cells, was counted using an inverted microscope (Nikon ECLIPS, E TS100, Japan).

### Animal studies

Four-week-old male BALB/C nude mice (SJA Experimental Animals Co., Ltd., Hunan, China) were housed in a controlled environment (25 °C, 95% humidity) within a clean-grade facility and acclimatized for 7 days prior to experimentation. To ensure unbiased group assignment, animals were systematically randomized into experimental cohorts using a computer-generated allocation sequence. For surgical procedures, mice were anesthetized via intraperitoneal injection of 0.5% pentobarbital sodium (40 mg/kg). Under aseptic conditions, a surgical microscope was used to guide a small incision through the skin and subcutaneous tissue, exposing the distal femoral metaphysis. A precision electric bone drill was then used to create a perpendicular cortical defect into the bone marrow cavity, ensuring minimal damage to surrounding tissues. After the injection, the cavity was sealed with bone wax, and the skin incision was sutured closed. Following a careful observation period of 4 weeks, bone destruction and infiltration of surrounding soft tissues were assessed and confirmed through microcomputed tomography (micro-CT; IVIS® Spectrum CT, PerkinElmer, MA, USA), thereby validating the successful establishment of the murine orthotopic tumor model for OS. Subsequently, the mice received intraperitoneal injections of DHT at a concentration of 1 mg/mL (50 μL) and/or (+)-JQ1 at a concentration of 4 mg/mL (50 μL), as dictated by the study design. Tumor growth in vivo and the expression levels of various markers of interest were evaluated utilizing a range of techniques, including micro-CT, western blotting, and mIF.

### Distribution of BRD4 and AR on chromatin detected by ChIP-Sequencing

OS cells were subjected to lysis and ultrasonic disruption both prior to and following treatment with 20 nM DHT, resulting in the generation of chromatin fragments ranging from 200 to 500 bp in length. Following this, chromatin fragments were immunoprecipitated using ChIP-grade antibodies against BRD4 and AR at a dilution of 1:100 (Abcam), in accordance with the manufacturer’s protocol. The resulting immunoprecipitated DNA was subsequently submitted to Novogene Bioinformatics Technology Co., Ltd. (Beijing, China) for genomic sequencing and bioinformatics analysis.

### Promoter activity examined by dual-luciferase reporter assay system

The full-length promoter sequences and the potential AR binding sites of PLCB4 were synthesized and cloned into a pmirGLO vector (Promega, Madison, WI, USA) to replace the original SV40 promoter. The vector was then transfected into OS cells, which were subsequently treated with DHT (20 nM) for 24 h. Subsequently, cells were then lysed and centrifuged, after which the supernatant was collected and placed on a multifunctional enzyme reader (Biolight Biotechnology Co., Ltd., Guangzhou, China) to determine the ratio of the luciferase activities between renilla and firey. The specific steps followed were conducted according to the kit’s operating instructions (Promega).

### Identification of binding motifs by electrophoretic mobility shift assay (EMSA)

Cytoplasmic and nuclear proteins were extracted from each cell group, and EMSA experiments were conducted in accordance with the manufacturer’s protocol provided in the LightShift® Chemiluminescent EMSA kit (Thermo Fisher Scientific). Specifically, AR proteins (Abcam), along with specific competitor probes and mutant competitor probes (Hippobio, Hangzhou, Zhejiang, China), were sequentially introduced based on the experimental grouping. This allowed the competitor probes to initially interact with the proteins, followed by the addition of labeled probes and a subsequent incubation at 25 °C for 30 min. A 6.5% EMSA gel was prepared using 0.5× TBE as the electrophoresis buffer, and pre-electrophoresis was conducted at 100 V for a duration of 30–60 min. Following this, 5 µL of 5× loading buffer was added, and the sample was promptly loaded onto the gel. Electrophoresis was carried out at 100 V until the bromophenol blue dye from the EMSA/Gel-Shift loading buffer migrated approximately one-quarter of the way up the gel, at which point electrophoresis was terminated. Finally, a wet transfer was executed using a transfer apparatus for 30–60 min, after which the process proceeded immediately to UV crosslinking, chemiluminescent detection, and visualization.

### Statistical analysis

Results were presented as mean ± SD, with *n* = 3, unless otherwise specified. While two-tailed unpaired Student’s *t*-test determined the statistical significance for two-group comparison, one-way ANOVA with Tukey or Dunnett was conducted for multiple-group comparison using GraphPad Prism 8 (GraphPad Software, Inc., San Diego, CA, USA). Pearson’s chi-squared test was performed to analyze the correlation between the expression of study indicators and patients’ situations that contain patients’ age, sex, and TNM stages. The correlation between the expression levels of BRD4, AR, and ERβ and the survival probabilities of OS patients was examined using linear regression analysis and the Kaplan–Meier (K–M) test, respectively. *P* < 0.05 were considered significant. Significance in all figures was indicated as follows: *P < 0.05, **P < 0.01, ***P < 0.001.

## Supplementary information

Supplemental figures

Original Data

Supplementary File

Supplement figureX1

## Data Availability

All data generated or analyzed in this study are included in this article. The datasets utilized and/or analyzed during the current research are available from the corresponding author upon reasonable request.
