# Supplementary material for: Androgen-induced AR-BRD4 transcriptional regulatory complex promotes malignant proliferation of osteosarcoma cells
Source: Cell Death Discov. 2025 Jun 10;11:272. doi: 10.1038/s41420-025-02541-6 (PMC12152148; doi:10.1038/s41420-025-02541-6)
Supplement: Supplementary file 1 — Supplemental figures [file 41420_2025_2541_MOESM1_ESM.pdf]

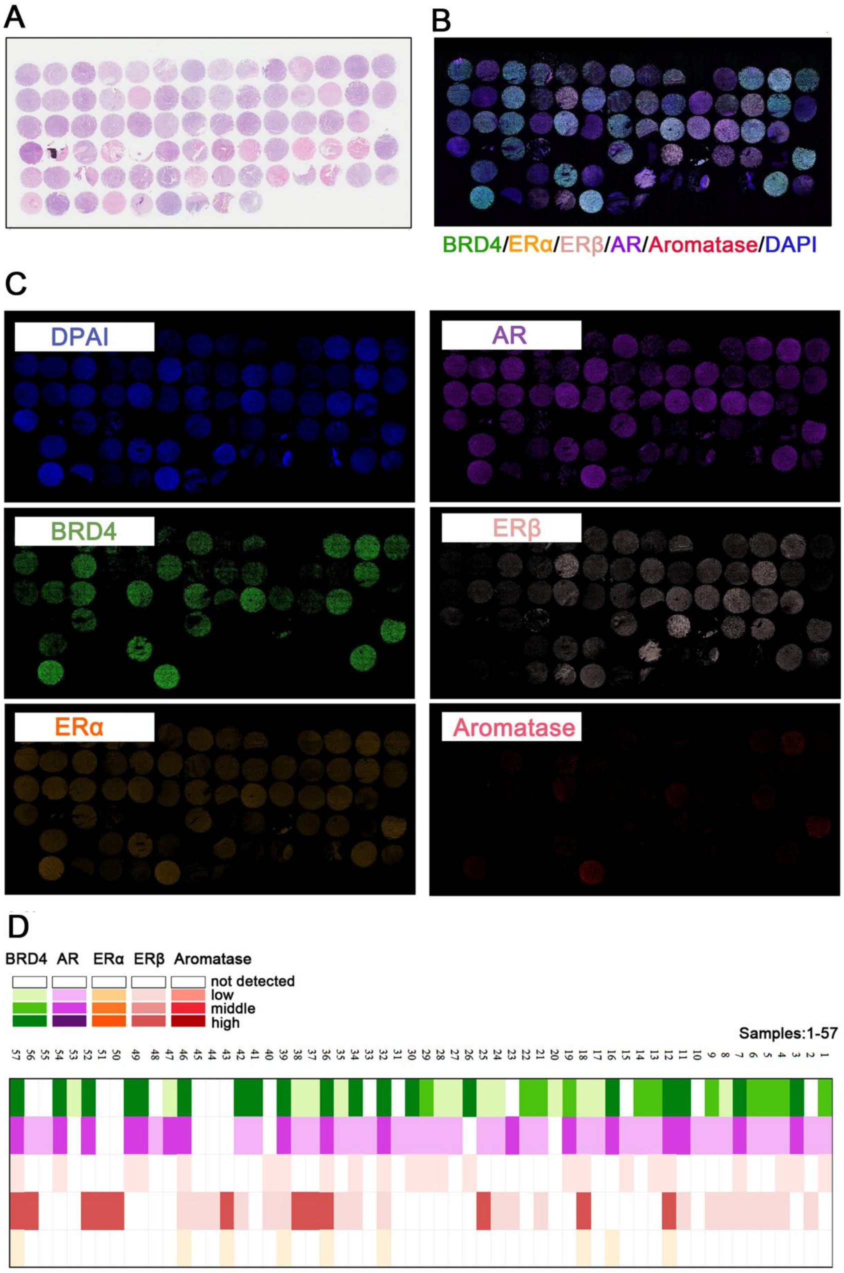

Supplemental figure 1. BRD4, AR, and ERβ exhibit relatively high positive rates in the tissues of OS patients. (A) H&E staining of the human OS TMA. (B) Merge image of five-color fluorescence labeling of BRD4 (green), AR (purple), ERα (orange), ERβ (pink), and aromatase (red) via the TSA experimental technique. (C) Single-color fluorescence results of various staining markers and cell nuclei (DAPI). (D) Expression analysis of BRD4, AR, ERα, ERβ, and aromatase in the OS TMA (57 samples available for analysis), with darker colors indicating higher positive expression rates.

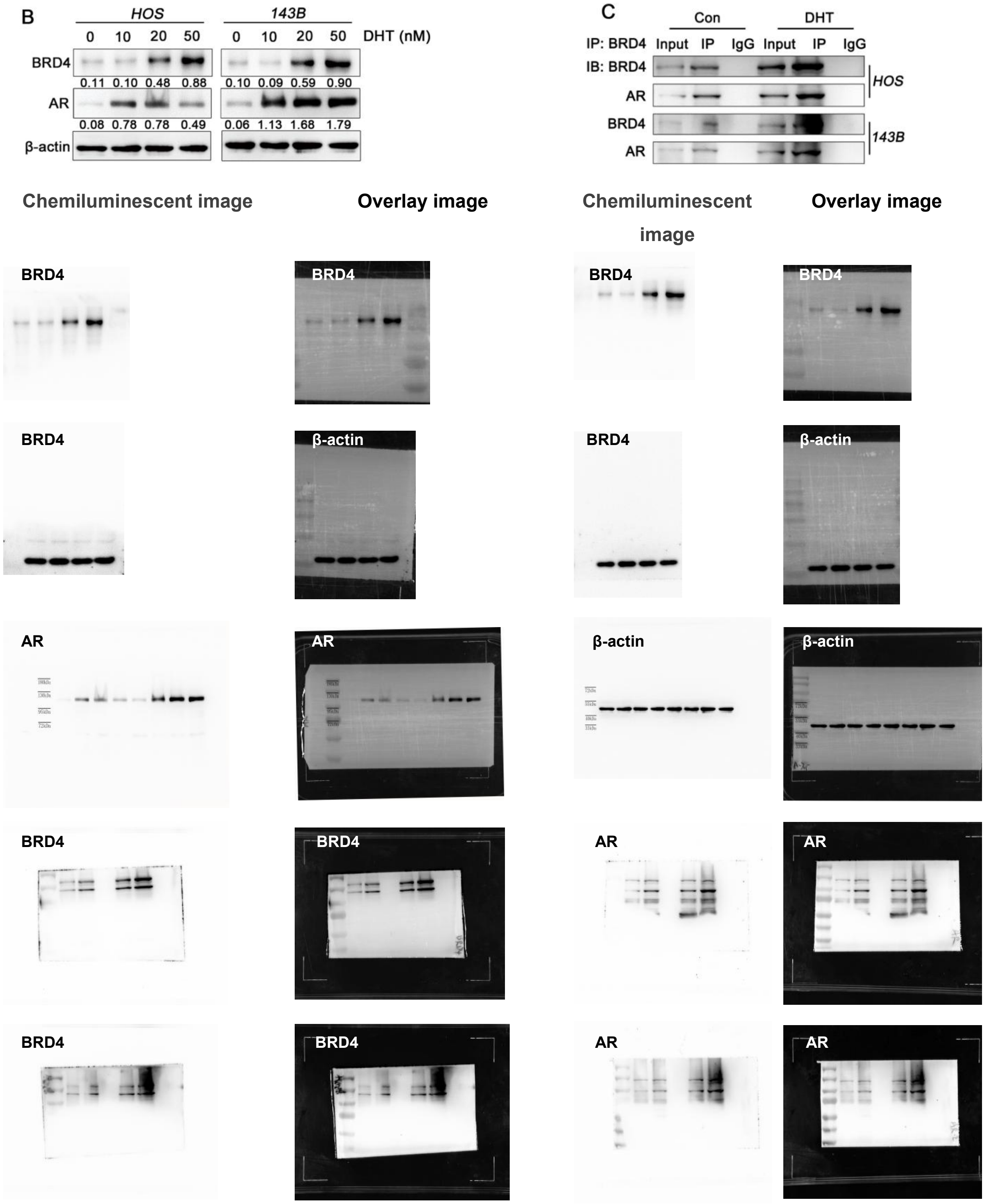

Supplemental figure 2. Original data of western blots for Fig.3B-C.

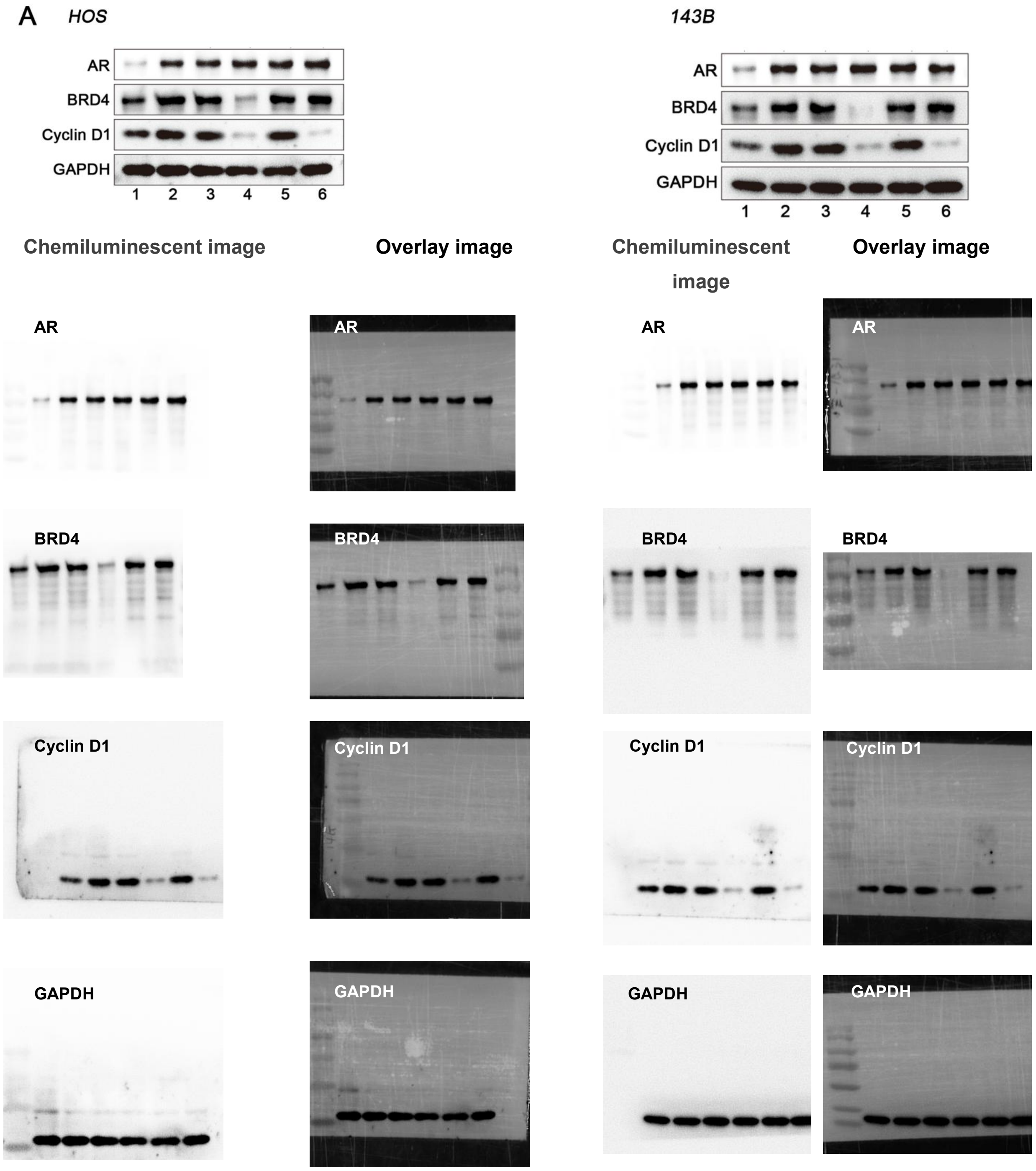

Supplemental figure 3. Original data of western blots for Fig.4A.

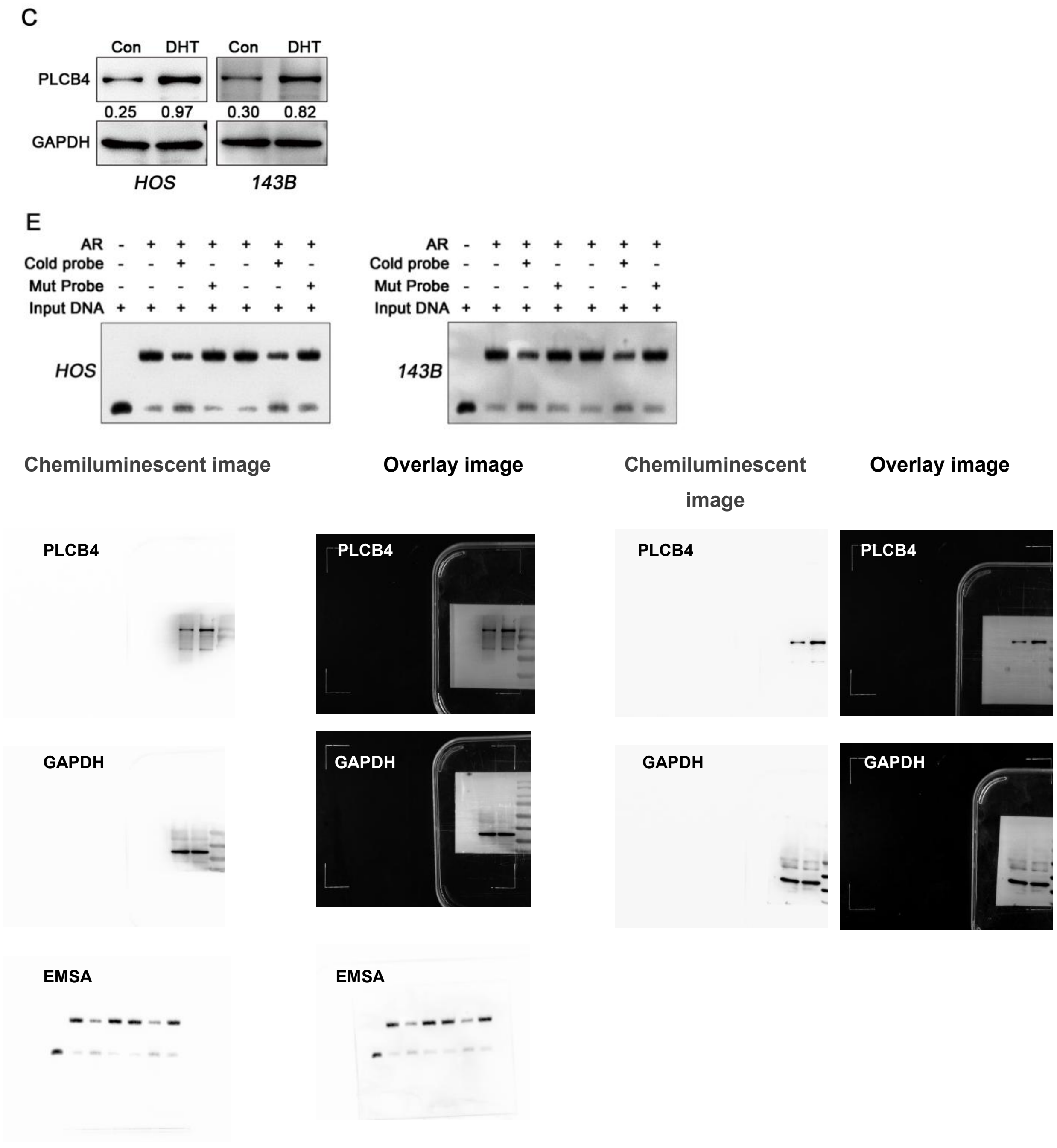

Supplemental figure 4. Original data of western blots for Fig.8C & E.

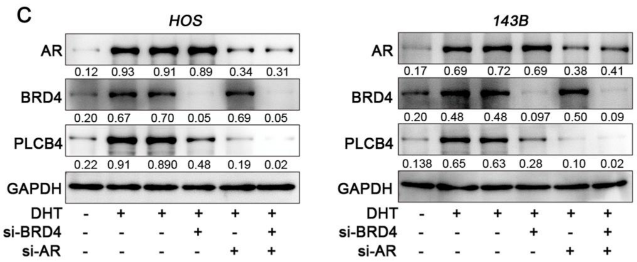

Chemiluminescent image | **Overlay image** | **Chemiluminescent image** | **Overlay image**

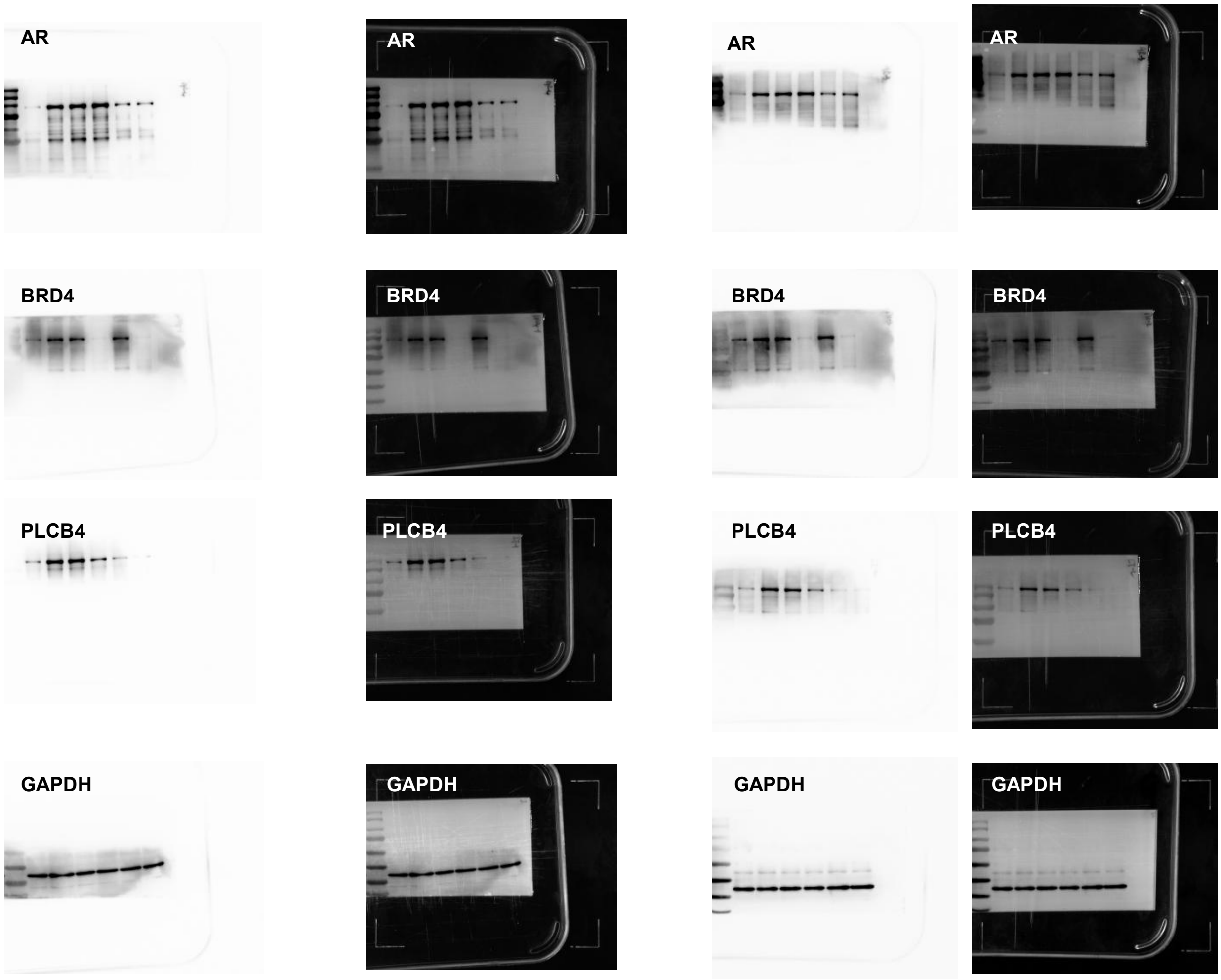

Supplemental figure 5. Original data of western blots for Fig.9C.

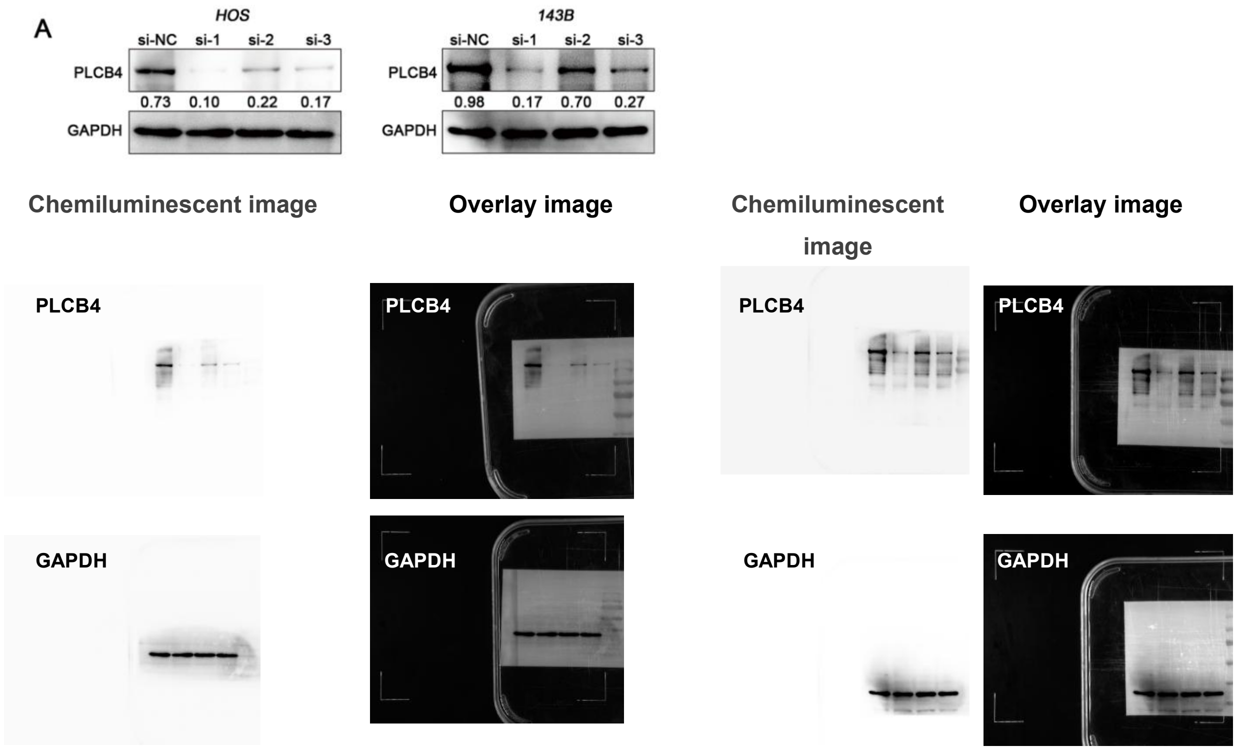

Supplemental figure 6. Original data of western blots for Fig.10A.

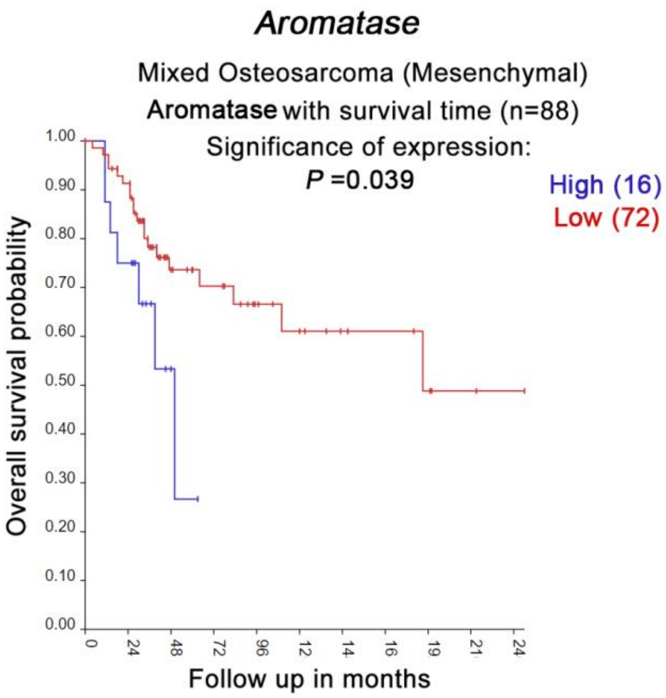

Supplemental figure 7. Statistical analysis of the expression level of aromatase and its association with the survival period of OS patients. Data obtained from the GEO database (GSE42352, n=88). K-M test and Log-rank test.
