# Supplementary material for: Androgen-induced AR-BRD4 transcriptional regulatory complex promotes malignant proliferation of osteosarcoma cells
Source: Cell Death Discov. 2025 Jun 10;11:272. doi: 10.1038/s41420-025-02541-6 (PMC12152148; doi:10.1038/s41420-025-02541-6)

Original data of western blots for Fig.3B-C

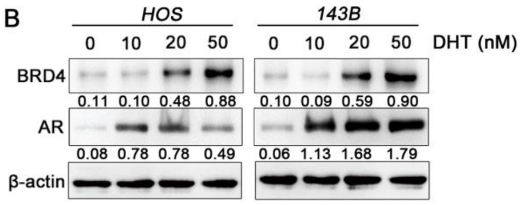

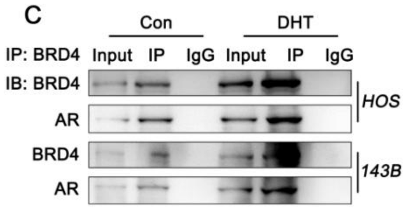

Chemiluminescent image **Overlay image** **Chemiluminescent image** **Overlay image**

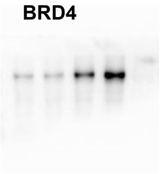

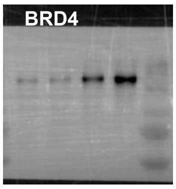

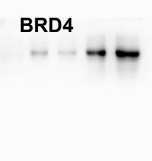

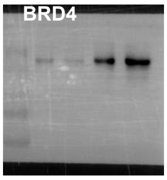

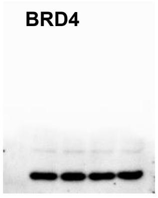

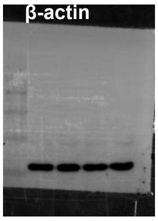

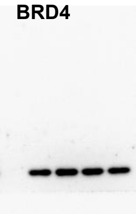

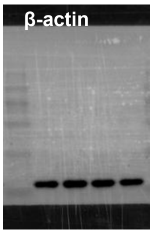

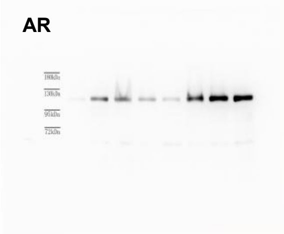

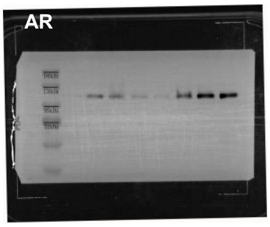

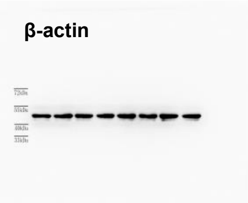

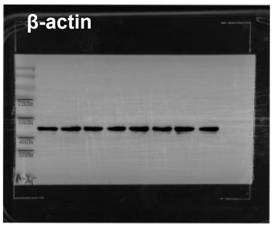

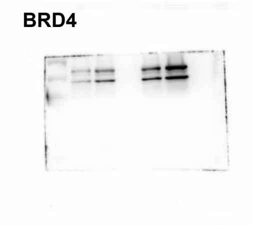

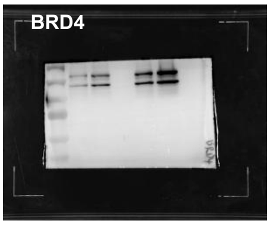

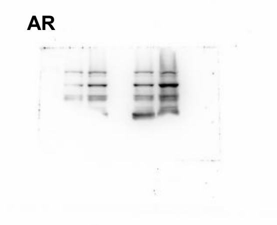

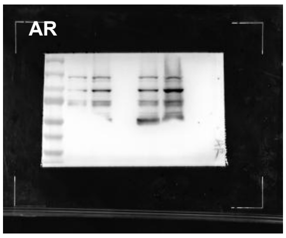

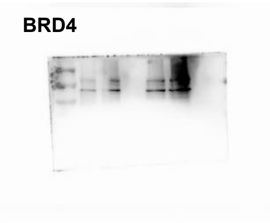

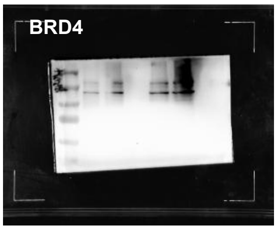

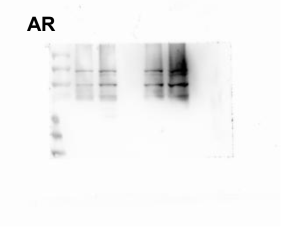

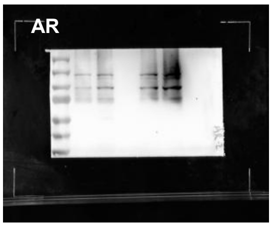

Original data of western blots for Fig.4A

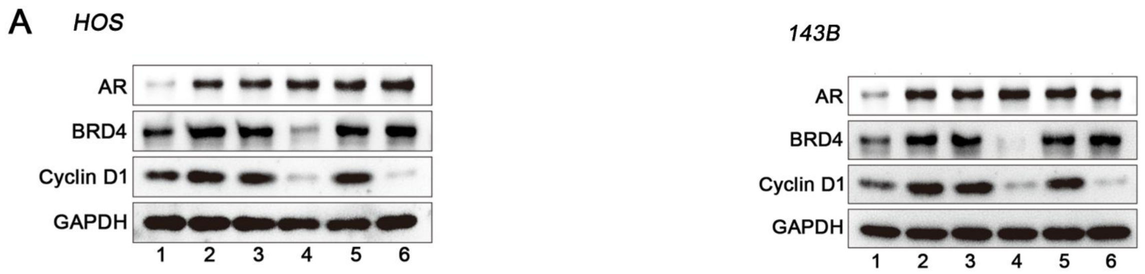

Chemiluminescent image | Overlay image | Chemiluminescent image | Overlay image

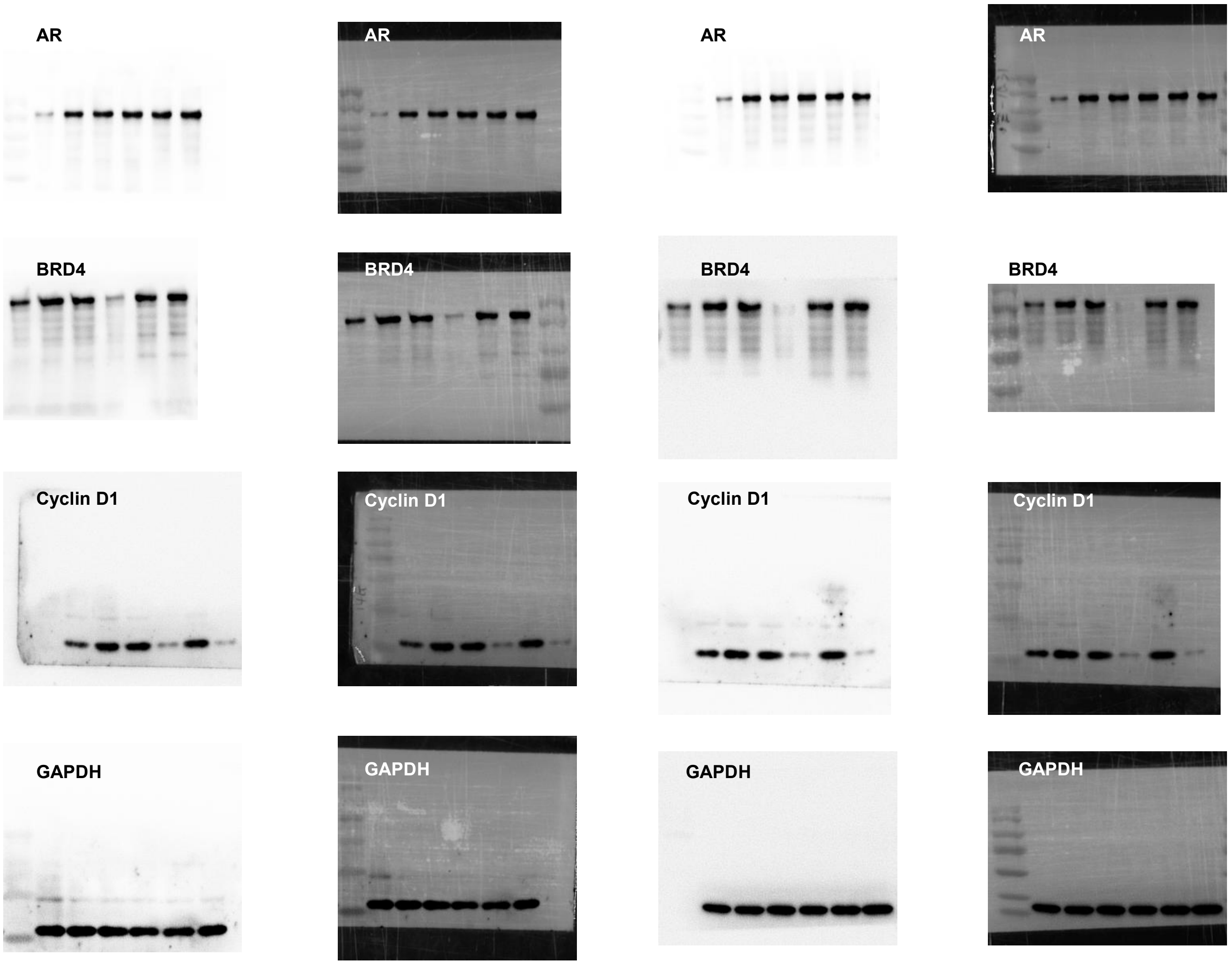

Original data of western blots for Fig.8C & E

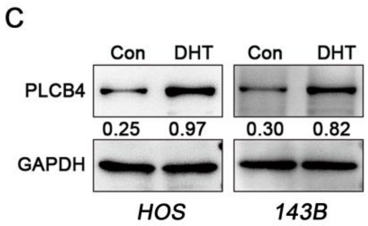

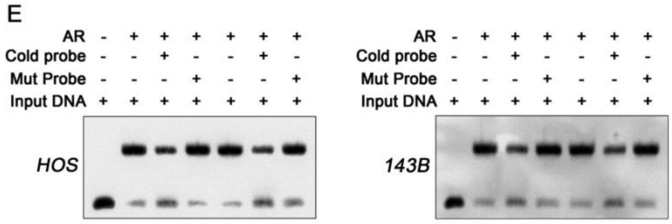

Chemiluminescent image | **Overlay image** | **Chemiluminescent image** | **Overlay image**

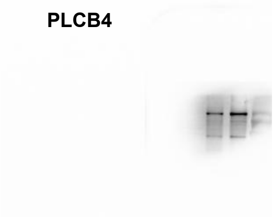

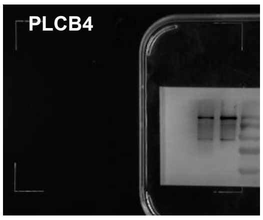

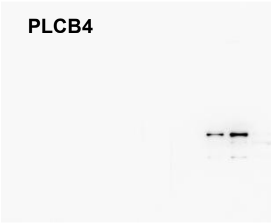

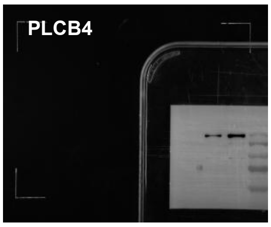

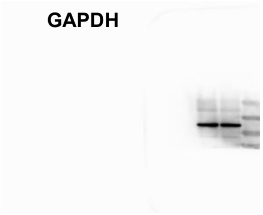

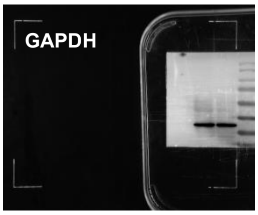

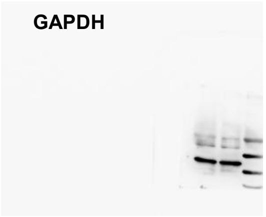

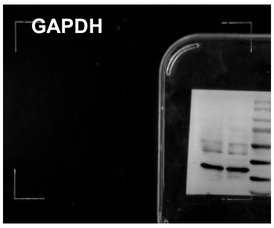

Original data of western blots for Fig.9C

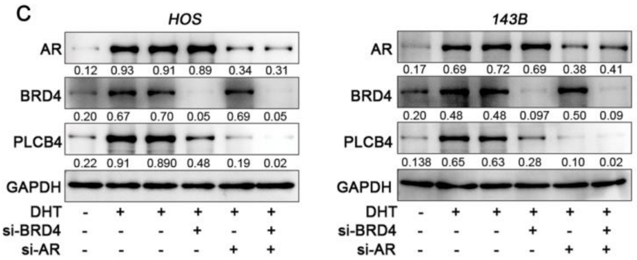

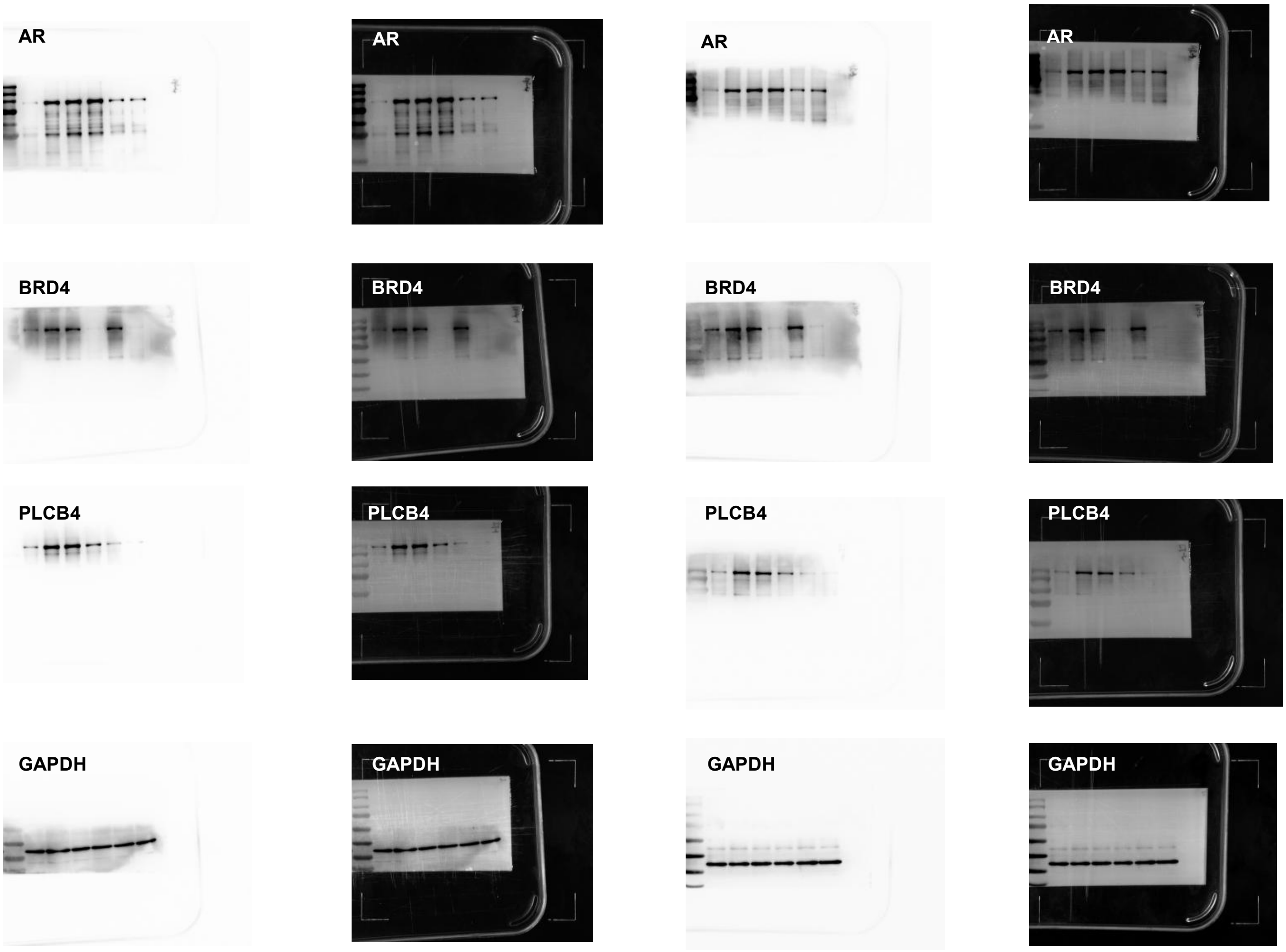

Original data of western blots for Fig.10A

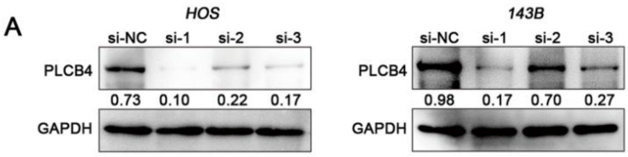

Chemiluminescent image | Overlay image | Chemiluminescent image | Overlay image

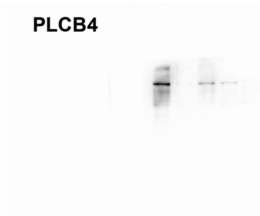

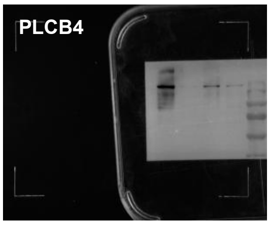

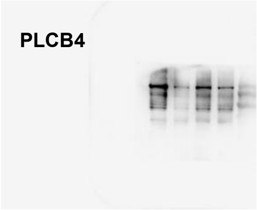

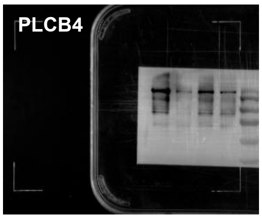

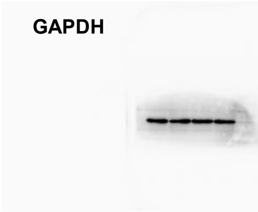

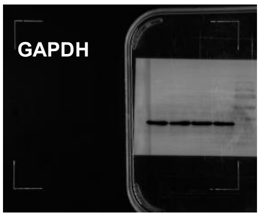

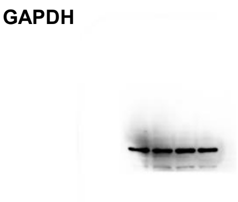

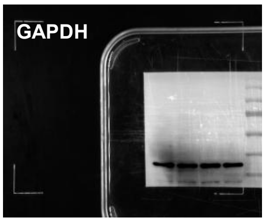

Supplement: Supplementary file 2 — Original Data [file 41420_2025_2541_MOESM2_ESM.pdf]
