# Supplementary figures and images for: Androgen-induced AR-BRD4 transcriptional regulatory complex promotes malignant proliferation of osteosarcoma cells
Source: Cell Death Discov. 2025 Jun 10;11:272. doi: 10.1038/s41420-025-02541-6 (PMC12152148; doi:10.1038/s41420-025-02541-6)

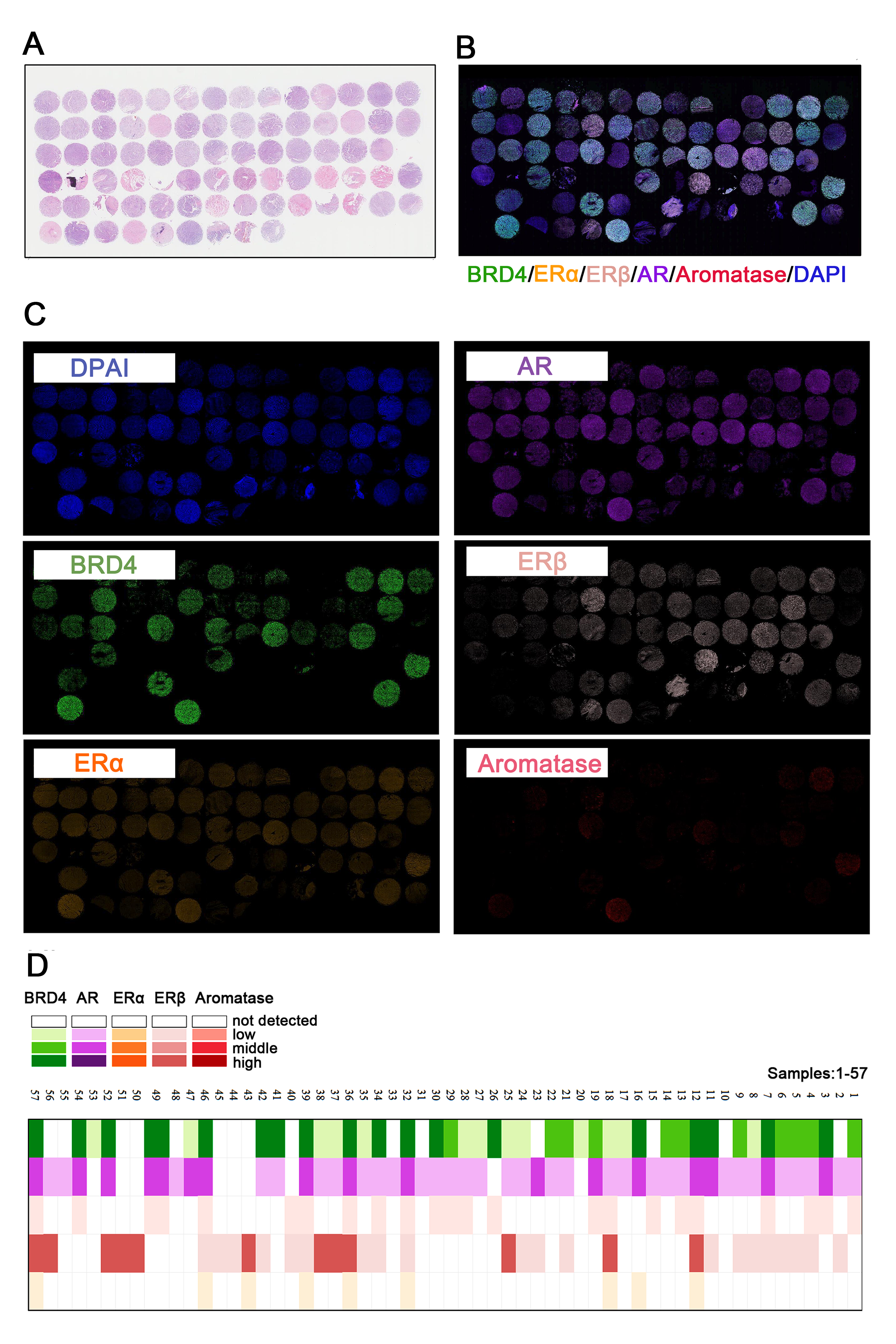

Supplement: Supplementary file 4 — Supplement figureX1 [file 41420_2025_2541_MOESM4_ESM.tif]
